# In vivo whole-cortex marker of excitation-inhibition ratio indexes cortical maturation and cognitive ability in youth

**DOI:** 10.1073/pnas.2318641121

**Published:** 2024-05-30

**Authors:** Shaoshi Zhang, Bart Larsen, Valerie J. Sydnor, Tianchu Zeng, Lijun An, Xiaoxuan Yan, Ru Kong, Xiaolu Kong, Ruben C. Gur, Raquel E. Gur, Tyler M. Moore, Daniel H. Wolf, Avram J. Holmes, Yapei Xie, Juan Helen Zhou, Marielle V. Fortier, Ai Peng Tan, Peter Gluckman, Yap Seng Chong, Michael J. Meaney, Gustavo Deco, Theodore D. Satterthwaite, B. T. Thomas Yeo

**Affiliations:** ^a^Centre for Sleep and Cognition and Centre for Translational Magnetic Resonance Research, Yong Loo Lin School of Medicine, National University of Singapore, Singapore 117594, Singapore; ^b^Department of Electrical and Computer Engineering, National University of Singapore, Singapore 117583, Singapore; ^c^N.1 Institute for Health, National University of Singapore, Singapore 117456, Singapore; ^d^Integrative Sciences and Engineering Programme, National University of Singapore, Singapore 119077, Singapore; ^e^Department of Medicine, Human Potential Translational Research Programme & Institute for Digital Medicine, Yong Loo Lin School of Medicine, National University of Singapore, Signapore 117456, Signapore; ^f^Penn Lifespan Informatics and Neuroimaging Center, University of Pennsylvania, Philadelphia, PA 19104; ^g^Department of Psychiatry, University of Pennsylvania, Philadelphia, PA 19104; ^h^Lifespan Brain Institute of Penn Medicine and Children's Hospital of Philadelphia, University of Pennsylvania, Philadelphia, PA 19104; ^i^Department of Pediatrics, University of Minnesota, Minneapolis, MN 55455; ^j^ByteDance, Singapore 048583, Singapore; ^k^Department of Radiology, University of Pennsylvania, Philadelphia, PA 19104; ^l^Department of Psychiatry, Brain Health Institute, Rutgers University, Piscataway, NJ 07103; ^m^Wu Tsai Institute, Yale University, New Haven, CT 06520; ^n^Department of Diagnostic and Interventional Imaging, Kandang Kerbau Women’s and Children’s Hospital, Singapore 229899, Singapore; ^o^Singapore Institute for Clinical Sciences, Agency for Science, Technology and Research, Singapore 138632, Singapore; ^p^Department of Diagnostic Radiology, Yong Loo Lin School of Medicine, National University of Singapore, Singapore 119074, Singapore; ^q^Centre for Human Evolution, Adaptation and Disease, Liggins Institute, University of Auckland, Auckland 1142, New Zealand; ^r^Department of Obstetrics and Gynaecology, Yong Loo Lin School of Medicine, National University of Singapore, Singapore 119228, Singapore; ^s^Department of Neurology and Neurosurgery, McGill University, Montreal, QC H3A1A1, Canada; ^t^Center for Brain and Cognition, Department of Technology and Information, Universitat Pompeu Fabra, Barcelona 08002, Spain; ^u^Institució Catalana de la Recerca i Estudis Avançats, Universitat Barcelona, Barcelona 08010, Spain; ^v^Martinos Center for Biomedical Imaging, Massachusetts General Hopstial, Charlestown, MA 02129

**Keywords:** default mode network, control network, neurodevelopment, cognition, resting state functional connectivity

## Abstract

A balanced excitation-inhibition ratio (E/I ratio) is critical for healthy brain function. Normative development of cortex-wide E/I ratio remains unknown. Here, we noninvasively estimate a putative marker of whole-cortex E/I ratio by fitting a large-scale biophysically plausible circuit model to resting-state functional MRI (fMRI) data. We first confirm that our model generates realistic brain dynamics in the Human Connectome Project. Next, we show that the estimated E/I ratio marker is sensitive to the gamma-aminobutyric acid (GABA) agonist benzodiazepine alprazolam during fMRI. Alprazolam-induced E/I changes are spatially consistent with positron emission tomography measurement of benzodiazepine receptor density. We then investigate the relationship between the E/I ratio marker and neurodevelopment. We find that the E/I ratio marker declines heterogeneously across the cerebral cortex during youth, with the greatest reduction occurring in sensorimotor systems relative to association systems. Importantly, among children with the same chronological age, a lower E/I ratio marker (especially in the association cortex) is linked to better cognitive performance. This result is replicated across North American (8.2 to 23.0 y old) and Asian (7.2 to 7.9 y old) cohorts, suggesting that a more mature E/I ratio indexes improved cognition during normative development. Overall, our findings open the door to studying how disrupted E/I trajectories may lead to cognitive dysfunction in psychopathology that emerges during youth.

## Significance

Healthy brain function requires a delicate balance of neural excitation (E) and inhibition (I). In animals, this balance—the E/I ratio—is known to decrease with the maturation of inhibitory circuitry during healthy development. However, in humans, the normative development of cortex-wide E/I ratio remains unclear. Here, we use a biophysical model and noninvasive brain scans to estimate a marker of E/I ratio. Spatial changes in our E/I ratio marker are consistent with a drug that decreases E/I ratio. We also find that our cortex-wide E/I ratio marker decreases during development. Furthermore, North American and Asian children with lower E/I ratio, especially in higher-order cortex, have better cognitive performance. Overall, the E/I ratio is a potential index of healthy neurocognitive development.

Healthy brain function requires a delicate balance between neural excitation (E) and inhibition (I) (1–4). This balance—the E/I ratio—is refined during critical developmental periods of heightened experience-dependent plasticity (5, 6). E/I imbalances during critical developmental periods are thought to contribute to the etiology of many psychiatric disorders (7, 8) and confer vulnerability to cognitive deficits (9, 10). Here, we capitalize on advances in biophysically plausible large-scale circuit models to chart the normative development of cortex-wide E/I ratio and uncover links to cognition.

Human cortical development unfolds hierarchically—sensory systems mature earlier, while association systems follow a more protracted developmental course extending through adolescence (11, 12). A potential mechanism driving this hierarchical development might be the temporal progression of critical plasticity periods along the sensorimotor-to-association axis (13–16). More specifically, the maturation of GABAergic inhibitory circuitry involving parvalbumin positive (PV) interneurons suppresses stimulus-irrelevant activity, yielding a higher signal-to-noise ratio (13). The maturation of PV interneurons also modulates long-term potentiation by enforcing a narrower spike integration window (17). Overall, the maturation of the inhibitory circuitry facilitates the experience-dependent pruning of excitatory pyramidal neuronal connections via the Hebbian mechanism, triggering a critical plasticity period (18–20). Therefore, a hallmark feature of the critical period development is a reduction in the E/I ratio (21, 22). While the hierarchical progression of inhibitory development is documented in animal models (14, 23), it is unclear whether the same mechanisms exist in humans, extend to the evolutionarily expanded association cortex, and impact cognitive ability.

Studying E/I ratio development in vivo in humans is challenging due to limitations in noninvasive neuroimaging techniques. MR spectroscopy studies suggest changes in the balance of excitatory and inhibitory neurotransmitter levels in single brain regions during development (24, 25). A recent study used a machine learning marker trained with pharmacological-functional MRI data to provide evidence of E/I ratio reduction in the association cortex during development (26). However, these past studies were limited to partial portions of the cortex, so normative development of cortex-wide E/I ratio remains unclear. Indirect estimates of whole-cortex E/I ratio have provided insights into autism spectrum disorder in adults and Alzheimer’s Disease (27–29), but these approaches mostly lack a direct mapping to an underlying biophysically plausible model of excitatory and inhibitory dynamics.

Biophysically plausible large-scale circuit models of coupled brain regions have provided mechanistic insights into spontaneous brain dynamics (30–32). However, most large-scale circuit models assume that local synaptic properties are spatially uniform across brain regions (9, 27, 33), which lacks biological plausibility. Indeed, spatial heterogeneity in excitatory and inhibitory cell types (34–36) might be a driver of large-scale brain dynamics (37, 38). Studies have shown that incorporating spatial heterogeneity across local synaptic parameters generates more realistic spontaneous brain dynamics (39, 40). Our previous study (41) demonstrated that parameterizing local synaptic parameters with anatomical and functional gradients led to dramatically more realistic brain dynamics in adults. However, we utilized a large-scale circuit model (42) that did not differentiate among excitatory and inhibitory neural populations, so the E/I ratio could not be derived.

Here, we investigate the development of cortical E/I ratio over youth and its association with cognitive ability. We apply our previous approach (41) to the feedback inhibition control (FIC) model with coupled excitatory and inhibitory neuronal populations (33). The resulting parameteric FIC (pFIC) model is used to derive a potential marker of E/I ratio. We first confirm that the pFIC model yields realistic brain dynamics in healthy young adults from the Human Connectome Project (HCP; 43). Using a pharmacological fMRI dataset (44), we show that the E/I ratio marker is sensitive to E/I ratio reduction induced by the GABA-agonist alprazolam. Then, using the Philadelphia Neurodevelopmental Cohort (PNC; 45, 46), we find that the E/I ratio declines across the cortex during youth. Furthermore, a lower E/I ratio indexes greater cognitive ability, with the strongest relationships observed in association cortex. We generalize the link between E/I ratio and cognitive ability in a younger GUSTO (Growing Up in Singapore with Healthy Outcomes) cohort (47). Overall, our study suggests that E/I ratio maturation might be a driver of healthy neurocognitive development during youth.

## 1. Results

### 1.1. Overview.

**Fig. 1.**
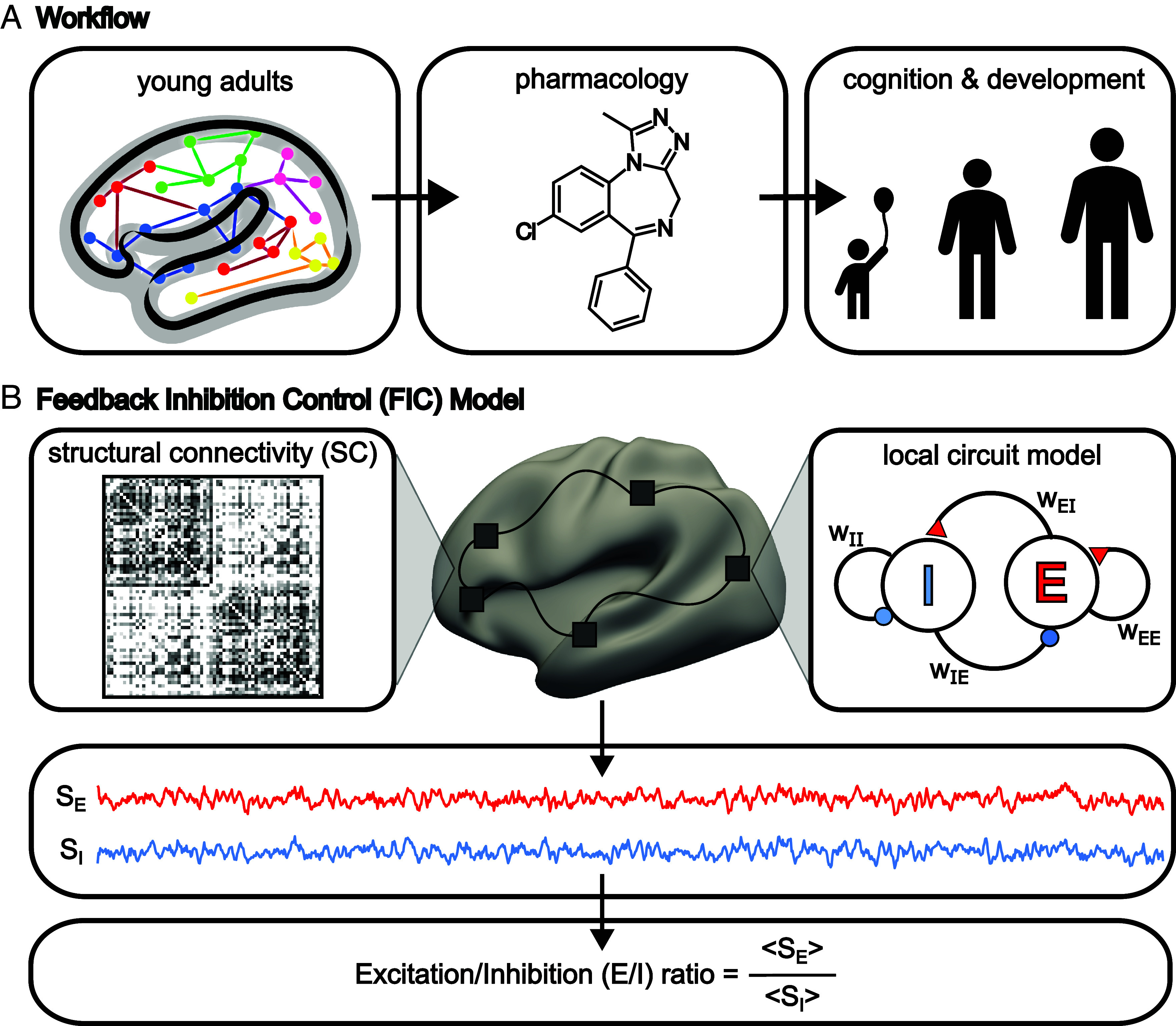
Workflow and schematic of the pFIC model. (*A*) Young adults from the HCP were used to evaluate the optimization of the spatially heterogeneous pFIC model. Pharmacological fMRI with benzodiazepine alprazolam was then used to evaluate the biological plausibility of the estimated E/I ratio. Next, the pFIC model was used to investigate the development of cortex-wide E/I ratio and its association with cognitive ability in the PNC dataset. Cognitive associations were replicated in a sample of 7-y-olds from the GUSTO cohort. HCP logo is used with permission from the HCP team. (*B*) The FIC model (33) is a neural mass model obtained by mean field reduction of spiking neuronal network models. The FIC model consists of differential equations at each cortical region governing the neural dynamics of excitatory and inhibitory neuronal populations (“E” and “I” respectively in the *Right* panel). A red triangle indicates an excitatory connection. A blue circle indicates an inhibitory connection. “w_xy_” indicates the connection strength from neuronal population x to neuronal population y. For example, “w_IE_” indicates the connection strength from the inhibitory population to the excitatory population. The regional models are connected by excitatory connections parameterized by a SC matrix. For a given set of model parameters, time courses of excitatory (S_E_) and inhibitory (S_I_) synaptic gating variables (representing the fraction of open channels) can be simulated. The E/I ratio was defined as the ratio between the temporal average of S_E_ and S_I_. Local synaptic parameters were estimated using the same approach as our previous study (41). We refer to the resulting model as the pFIC model.

We first evaluated the optimization of the spatially heterogeneous pFIC model in the HCP dataset (Fig. 1*A*). The biological plausibility of the estimated marker of E/I ratio was then evaluated using pharmacological fMRI involving GABAergic benzodiazepine alprazolam. Finally, we investigated developmental changes and cognitive effects of E/I ratio in the PNC dataset. Assocations with cognition were replicated in the GUSTO cohort.

### 1.2. Optimization of the pFIC Model.

We randomly divided 1004 HCP participants (43, 48) into 3 nonoverlapping training (N = 335), validation (N = 335), and test (N = 334) sets. The Desikan–Killiany anatomical parcellation (49) with 68 cortical regions of interest (ROIs) was used to generate group-level structural connectivity (SC), static functional connectivity (FC), and FC dynamics (FCD) from the training, validation, and test sets separately. To compute FCD for each fMRI run, a 68 × 68 FC matrix was computed for each sliding window of length ~60 s. The 68 × 68 FC matrices were then correlated across the 1118 windows, yielding a 1118 × 1118 FCD matrix (41). The FCD matrix has been shown to reflect temporal fluctuations in resting-state FC that are not captured by static FC (50, 51). See *SI Appendix*, *Supplementary Methods S2* for details.

The FIC model (33) is a neural mass model obtained by mean field reduction of a spiking neuronal network model (52, 53). The model comprises ordinary differential equations (ODEs) at each cortical region describing the dynamics of excitatory and inhibitory neuronal populations (Fig. 1 *B*, *Top*). The local dynamics are driven by recurrent connections within separate excitatory and inhibitory populations, as well as connections between excitatory and inhibitory populations. Greater excitatory-to-excitatory recurrent strength (w_EE_) and smaller inhibitory-to-excitatory connection strength (w_IE_) amplify synaptic currents of the excitatory population. Similarly, greater excitatory-to-inhibitory connection strength (w_EI_) and smaller inhibitory-to-inhibitory recurrent strength (w_II_) amplify synaptic currents of the inhibitory population. Neuronal noise in each cortical region is controlled by the noise amplitude σ. Finally, the excitatory populations of the regional local models are connected via the SC matrix, scaled by a global constant *G*.

Following previous studies (33, 39), w_II_ was set to one and w_IE_ was automatically set to maintain a uniform baseline excitatory firing rate of around 3 Hz. Excitatory-to-excitatory recurrent strength (w_EE_), excitatory-to-inhibitory connection strength (w_EI_), regional noise amplitude (σ) and the SC scaling constant (*G*) were estimated using our previous approach (41). More specifically, w_EE_, w_EI_, and σ were parameterized as a linear combination of the principal resting-state FC gradient (54) and T1w/T2w myelin estimate (55), resulting in 9 unknown linear coefficients and 1 unknown parameter *G*. We refer to the resulting model as the pFIC model.

The 10 pFIC parameters were estimated using the covariance matrix adaptation evolution strategy (CMA-ES) (56) by minimizing the difference between simulated and empirical fMRI data. More specifically, agreement between empirical and simulated FC matrices was defined as the Pearson’s correlation (*r*) between the *z*-transformed upper triangular entries of the two matrices. Larger *r* indicates more similar static FC. However, Pearson’s correlation does not account for scale difference, so we also computed the absolute difference (*d*) between the means of the empirical and simulated FC matrices (39). A smaller *d* indicates more similar static FC. The inclusion of *d* was necessary to prevent overly synchronized fMRI signals (*SI Appendix*, Fig. S1). Finally, we do not expect the brain states of two participants to be the same at any given timepoint *t* during the resting state, i.e., there is no temporal correspondence between participants in the resting state. Because FCD was computed based on sliding window FC, there was similarly no temporal correspondence between simulated and empirical FCD matrices. Therefore, disagreement between the simulated and empirical FCD matrices was defined as the Kolmogorov–Smirnov (KS) distance, following previous studies (41, 50). The KS distance was defined as the maximum distance between the cumulative distribution functions obtained by collapsing the upper triangular entries of simulated and empirical FCD matrices, so no temporal correspondence was assumed (more details in *SI Appendix*, *Supplementary Methods S10*). The overall cost was defined as (1 – *r*) + *d* + *KS*. A smaller cost indicates better agreement between simulated and empirical fMRI.

### 1.3. The pFIC Model Generates Realistic fMRI Dynamics.

**Fig. 2.**
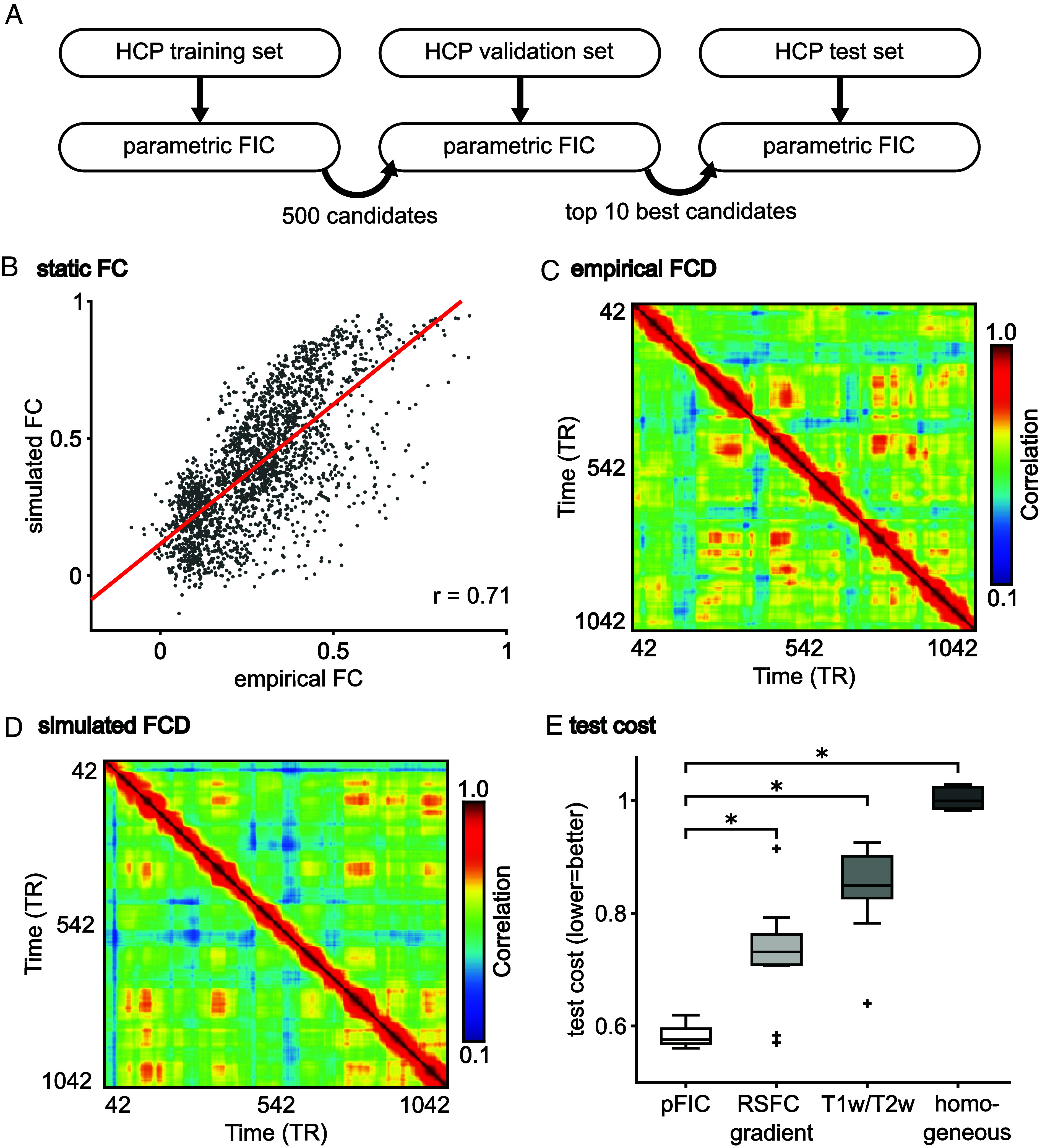
The pFIC model generates more realistic fMRI dynamics than the spatially homogeneous FIC model. (*A*) The CMA-ES algorithm (41, 56) was applied to the HCP training set to generate 500 sets of model parameters. The top 10 parameter sets from the validation set were evaluated in the test set. (*B*) Agreement (Pearson’s correlation) between empirical and simulated static FC in the HCP test set. (*C*) Empirical FCD from an HCP test participant. (*D*) Simulated FCD from the pFIC model using the best model parameters (from the validation set) and SC from the test set. (*E*) Total test cost of the pFIC model compared with three control conditions: 1) local synaptic parameters parameterized by only principal resting-state FC gradient, 2) local synaptic parameters parametrized by only T1w/T2w ratio map and 3) local synaptic parameters constrained to be spatially uniform. The boxes show the interquartile range (IQR) and the median. Whiskers indicate 1.5 IQR. Black crosses represent outliers. * indicates that the pFIC model achieved statistically better (lower) test cost.

We first demonstrate that the parametrization of the local synaptic parameters with T1w/T2w and FC gradient led to more realistic brain dynamics than spatially homogeneous parameters (Fig. 2*A*). We applied CMA-ES to the HCP training set to generate 500 candidate model parameter sets. The 500 parameter sets were evaluated in the HCP validation set. The top 10 parameter sets from the HCP validation set were used to simulate FC and FCD using SC from the HCP test set, which were then compared with empirical FC and FCD from the HCP test set. A strong agreement between simulated and empirical FC (as well as between simulated and empirical FCD) would suggest that the pFIC model was able to generate realistic brain dynamics.

Fig. 2*B* visualizes the correlation between empirical and simulated FC in the HCP test set (based on the best model parameters from the validation set). Across the 10 best parameter sets from the validation set, correlation between empirical and simulated static FC was 0.71 ± 0.005 (mean ± SD) in the test set. As a reference, correlation between empirical FC and SC in the test set was 0.48. On the other hand, the absolute difference between the means of the empirical and simulated FC matrices was 0.11 ± 0.015 in the test set. This suggests that the pFIC model was able to generate realistic FC.

Fig. 2*C* shows the empirical FCD from a single run of a representative HCP test participant. Fig. 2*D* shows the simulated FCD using the best model parameters (from the validation set) and SC from the test set. Off-diagonal red blocks in both empirical and simulated FCD indicated recurring FC patterns that were not simply due to temporal autocorrelation. Similarity in the amount of off-diagonal red blocks between empirical and simulated FCD suggests that the pFIC model was able to generate realistic FCD. Across the 10 best candidate sets from the validation set, the KS distance between empirical and simulated FCD was 0.18 ± 0.028 in the HCP test set. Disagreement between simulated and empirical fMRI appeared more pronounced in posterior regions, but the pattern of disagreement was not correlated with the RSFC gradient or the T1w/T2w ratio map (*SI Appendix*, Figs. S2 and S3).

Overall, the pFIC model was able to generate realistic FC and FCD, yielding an overall cost of 0.58 ± 0.018 in the HCP test set (Fig. 2*E*). Parameterizing model parameters with only the principal FC gradient or only T1w/T2w ratio map led to worse (higher) cost in the HCP test set (Fig. 2*E*). Most large-scale circuit model studies assume spatially homogeneous parameters. When local synaptic parameters (w_EE_, w_EI_, and σ) were constrained to be uniform across brain regions (33, 57) and optimized by CMA-ES, the cost was poor in the test set (Fig. 2*E*). These results emphasize the importance of parameterizing local synaptic parameters with spatial gradients that smoothly varied from sensory-motor to association cortex. Consistent with our previous study (41), the T1w/T2w and FC gradient were complementary in the sense that combining the two spatial maps led to more realistic fMRI dynamics.

### 1.4. Estimated E/I Ratio Is Sensitive to the Effect of Benzodiazepine Alprazolam.

In the previous section, we showed that the pFIC model could be effectively optimized to generate realistic fMRI dynamics. Here, we evaluated the biological plausibility of the estimated E/I ratio in a pharmacological-fMRI dataset (44) comprising 45 participants, who completed a placebo-controlled double-blind fMRI study with benzodiazepine alprazolam. Alprazolam is a benzodiazepine that enhances GABAergic signaling at GABA_A_ receptor subunits, including α_1,2,3,5_ and γ_1−3_ (58, 59). Alprazolam enhances GABAergic inhibitory signaling via positive allosteric modulation, thus reducing the E/I ratio (60). Therefore, we hypothesized that the E/I ratio estimated with the pFIC model would be lower during the alprazolam condition compared with the placebo condition.

The 45 participants were equally divided into training, validation, and test sets. Group-level SC, first principal FC gradient, and T1w/T2w ratio maps from the HCP dataset were used in the following analysis. For each experimental condition (placebo or alprazolam), 250 candidate parameter sets were generated from the condition’s training set. The top 10 parameter sets from the validation set were evaluated in the test set. The costs of the 10 parameter sets generalized well to the test set (*SI Appendix*, Fig. S4), suggesting that there was no overfitting in the validation set.

One challenge in analyzing this dataset was that the fMRI data were acquired with a limited field of view. Therefore, 26 out of 68 Desikan-Killiany ROIs with less than 50% coverage (*SI Appendix*, Fig. S5) were not considered during the estimation of the model parameters. The estimated model parameters were extrapolated to the entire cortex (see *SI Appendix*, *Supplementary Methods S11* for details) and used to simulate the excitatory (S_E_) and inhibitory (S_I_) time courses (Fig. 1*B*). Motivated by rodent studies, the E/I ratio was defined as the ratio between the temporal average of S_E_ and S_I_ (61).

**Fig. 3.**
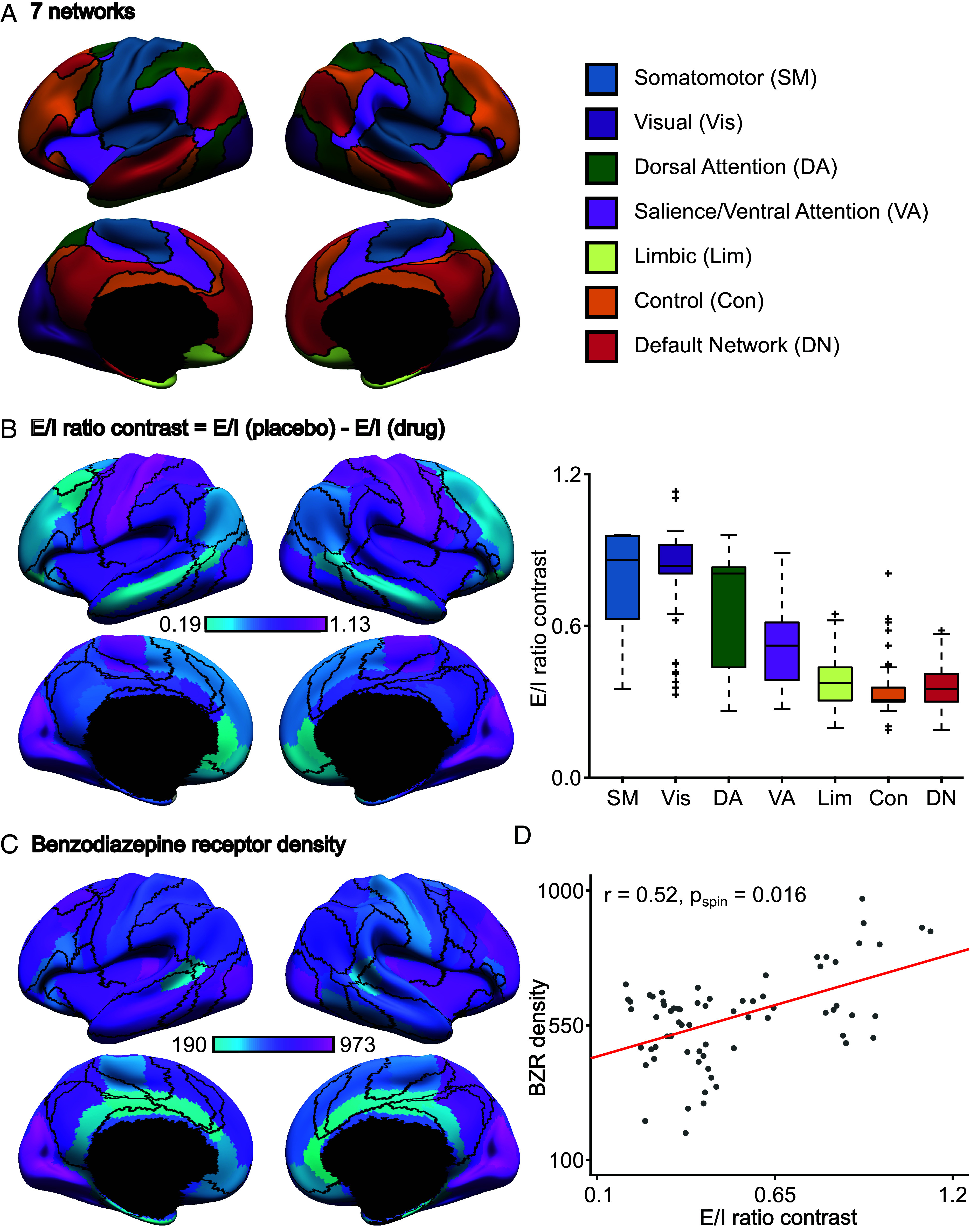
E/I ratio estimate is sensitive to the effect of benzodiazepine alprazolam. (*A*) Seven resting-state networks (62). (*B*) *Left*: Regional E/I ratio contrast overlaid with the boundaries (black) of seven resting-state networks. Sixty-seven out of 68 regions showed significant E/I ratio difference between placebo and drug sessions after FDR correction (*q* < 0.05). E/I ratio difference was greater than zero for all regions, consistent with lower E/I ratio during the alprazolam session. *Right*: E/I ratio differences exhibited a spatial gradient with higher differences in sensory-motor regions compared with regions in the control and default networks. The boxes show the interquartile range (IQR) and the median. Whiskers indicate 1.5 IQR. Black crosses represent outliers. (*C*) Spatial distribution of BZR density (pmol/mL) from in vivo positron emission tomography in a separate group of participants (63). (*D*) Higher regional BZR density was associated with larger E/I ratio changes during the drug session (*r* = 0.52, two-tail spin test *P* = 0.016).

An E/I ratio contrast was computed by subtracting the E/I ratio estimated during the drug (alprazolam) session from the E/I ratio estimated during the placebo session. Since alprazolam is expected to reduce the E/I ratio, we hypothesized that the E/I ratio would be lower during the alprazolam condition, yielding a positive E/I ratio contrast. Consistent with our hypothesis, the E/I ratio contrasts of all regions were positive (Fig. 3*B*). Sixty-seven out of 68 regions exhibited E/I ratio contrasts statistically different from zero after correcting for multiple comparisons with a false discovery rate (FDR) of *q* < 0.05. We note that there was no motion difference between the drug and placebo fMRI sessions (*P* > 0.1). These results suggest that the E/I ratio estimated by the pFIC model was sensitive to the pharmacological enhancement of inhibitory activities.

Since the distribution of benzodiazepine receptors (BZR) density is not spatially uniform (63), we hypothesized that the E/I ratio contrast would also not be spatially uniform, and would align with BZR density. Supporting this, we found that the E/I ratio contrast exhibited a spatial gradient with the strongest effects in sensory-motor networks and the weakest effects in control and default networks (Fig. 3*B*). Fig. 3*C* shows the spatial distribution of BZR density estimated from in vivo positron emission tomography in a separate group of participants (63). Regions with greater BZR density exhibited greater reduction in E/I ratio during the drug session (*r* = 0.52; two-tail spin test *P* = 0.016; Fig. 3*D*). Therefore, the spatial distribution of E/I ratio contrast was biologically plausible.

To evaluate robustness, we repeated the above analyses 5 times with different random splits of the 45 participants into training, validation, and test sets. The results were similar across the 5 splits (*SI Appendix*, Figs. S6 and S7). Results from the most representative split are shown in Fig. 3. Using this most representative split, we performed several additional sensitivity analyses. In the previous analyses, the acceptable excitatory firing rate was constrained to be between 2.7 Hz and 3.3 Hz. Relaxing the thresholds to between 2.5 Hz and 3.5 Hz yielded similar results (*SI Appendix*, Fig. S8). Changing the ROI coverage threshold from 50 to 60% also yielded similar results (*SI Appendix*, Fig. S9). We repeated the analysis using a 100-region homotopic functional parcellation (64), which also yielded similar results (*SI Appendix*, Fig. S10). Pairwise comparisons between the control analyses are found in *SI Appendix*, Fig. S11. Similar results were obtained with log-transformation or square root of BZR density (*SI Appendix*, Fig. S12).

### 1.5. The E/I Ratio Declines with Development in Youth.

Having demonstrated that the E/I ratio estimates were sensitive to the alprazolam-induced enhancement of inhibitory activities, we next explored how the E/I ratio changes during development in the PNC (45, 46). We hypothesized that the estimated E/I ratio would decline with age.

**Fig. 4.**
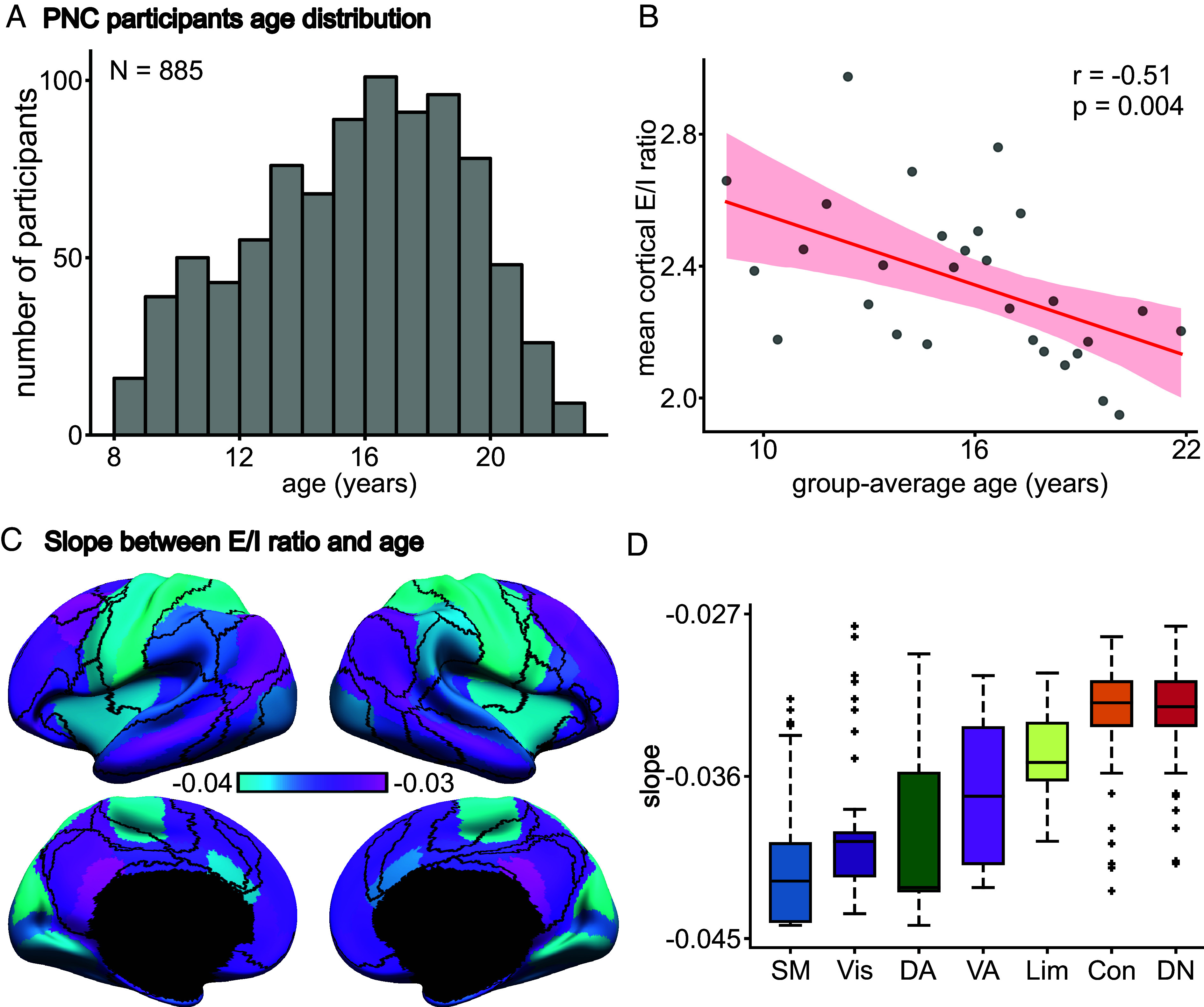
E/I ratio continuously declines throughout child and adolescent development. (*A*) Age distribution of 885 PNC participants (mean = 15.66, SD = 3.36, min = 8.17, max = 23). (*B*) Participants in older age groups exhibited lower E/I ratio (*r* = −0.51, *P* = 0.004). Participants were divided into 29 nonoverlapping age groups. There are 29 dots in the scatter plot, corresponding to the 29 age groups. The shaded area depicts 95% CI of the linear relationship. (*C*) Spatial distribution of linear regression slope between regional E/I ratio and age. The values represent the rate of E/I ratio changes during development. All slopes were negative and significant (FDR *q* < 0.05). (*D*) The slopes exhibited a spatial gradient with sensory-motor networks showing the fastest E/I ratio reduction and association networks showing slower E/I ratio reduction. The boxes show the interquartile range (IQR) and the median. Whiskers indicate 1.5 IQR. Black crosses represent outliers.

After data preprocessing and quality control, we obtained a sample of 885 participants aged 8 to 23 y (Fig. 4*A*). Participants were sorted according to age and evenly divided into 29 age groups, so each group comprised 30 or 31 participants. Within each age group, 15 participants were randomly selected as the validation set, while the remaining participants were assigned to the training set. For each age group, 250 candidate model parameter sets were generated from the group’s training set using CMA-ES and evaluated in the group’s validation set; the parameter set with the lowest validation cost was used to estimate the regional E/I ratio across the cortex.

We performed linear regression between age and mean cortical E/I ratio (i.e., E/I ratio averaged across the whole cortex), as well as between age and regional E/I ratio. Mean cortical E/I ratio declined throughout child and adolescent development (*r* = −0.51, *P* = 0.004; Fig. 4*B*). This E/I ratio reduction was statistically significant for all cortical regions (FDR *q* < 0.05; Fig. 4*C*). Furthermore, the rate of E/I ratio decrease exhibited a spatial gradient with sensory-motor regions exhibiting a greater rate of E/I ratio decrease (i.e., more negative slope) compared with association networks (Fig. 4*D*).

To evaluate the robustness of these effects, the PNC analyses were repeated 5 times with different splits of the participants (within each age group) into training and validation sets. The results were similar across the 5 random splits of the data (*SI Appendix*, Figs. S13 and S14). We conducted several additional sensitivity analyses using the most representitve split (which was shown in Fig. 4). Relaxing the firing rate thresholds to between 2.5 Hz and 3.5 Hz yielded similar results (*SI Appendix*, Fig. S15), as did using a 100-region homotopic parcellation (64) (*SI Appendix*, Fig. S16). Pairwise comparisons between the control analyses are found in *SI Appendix*, Fig. S17. Finally, consistent with the literature, younger participants exhibited higher head motion during the fMRI scan. Therefore, as a control analysis, we regressed out mean framewise displacement from the E/I ratio estimates of each age group, yielding similar results (*SI Appendix*, Fig. S18).

### 1.6. Lower E/I Ratio Is Associated with Better Cognition within the Same Age Group.

Having shown that older children exhibited lower E/I ratio (Fig. 4*B*), we next evaluated the cognitive implications of such a decline in the E/I ratio as part of normative development. We hypothesize that a lower E/I ratio would be associated with better cognition. To test this hypothesis, we compared the E/I ratio of PNC participants who were matched on age but differed in cognitive performance.

**Fig. 5.**
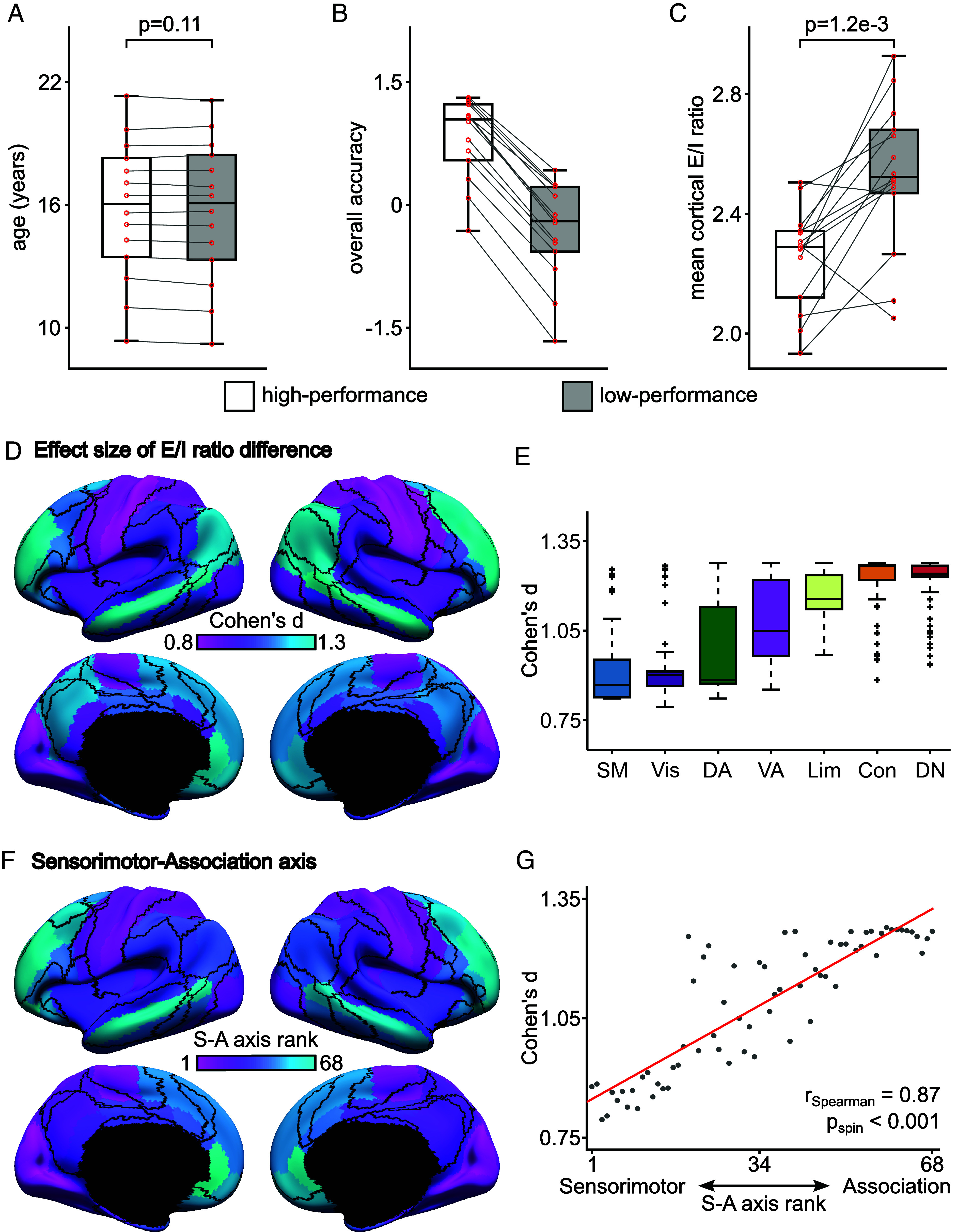
Lower E/I ratio is associated with better cognitive performance within the same age group in the PNC dataset. (*A*) Boxplots of age, (*B*) “overall accuracy,” and (*C*) mean cortical E/I ratio of high-performance and low-performance (overall accuracy) groups. The mean cortical E/I ratio of the high-performance group was significantly lower than that of the low-performance group (FDR *q* < 0.05). (*D*) Spatial distribution of effect size of regional E/I ratio difference between high-performance and low-performance groups. All regions were significant after FDR correction with *q* < 0.05. (*E*) Effect size of E/I ratio differences in cognition is larger in control and default networks compared with sensorymotor regions. The boxes show the interquartile range (IQR) and the median. Whiskers indicate 1.5 IQR. Black crosses represent outliers. (*F*) ROI rankings based on the S-A axis (66). Lower ranks were assigned to ROIs that were more toward the sensorimotor pole; higher ranks were assigned to ROIs that were more toward the association pole. (*G*) Agreement between effect size of E/I ratio difference and S-A axis rank. Spearman’s correlation *r* = 0.87, two-tailed spin test *p* < 0.001.

Participants in the PNC completed the Penn Computerized Neurocognitive Battery, a 12-task battery that has been previously summarized using an overall (domain-general) measure of accuracy as well as three domain-specific factor scores (65). Participants were divided into 14 high-performance groups and 14 low-performance groups based on the overall accuracy measure. Each high-performance group was age-matched to a low-performance group (Fig. 5*A*). Each low-performance or high-performance group comprised 31 or 32 participants. For each group, 15 participants were randomly assigned to the validation set, while the remaining participants were assigned to the training set. For each group, 250 candidate parameter sets were generated from the training set and the top parameter set from the validation set was used to estimate the E/I ratio; we compared the E/I ratio between the high- and low-performance groups.

The high-performance group exhibited a lower mean cortical E/I ratio than the low-performance group (two-tailed t test *P* = 1.2 × 10^−3^; Fig. 5*C*). There was no motion difference between high-performance and low-performance groups (*P* > 0.2). To test for domain specificity, we also compared the E/I ratio for the three domain-specific factor scores (complex reasoning, memory, and social cognition), but observed no statistical difference after correcting for multiple comparisons (*SI Appendix*, Fig. S19).

Having found global differences in the E/I ratio between the high and low cognitive performance groups, we next evaluated regional effects (Fig. 5*D*). We found that E/I ratio differences between low-performance and high-performance groups were larger in control and default networks, compared with sensory-motor regions (Fig. 5*E*; all FDR *q* <0.05). Notably, the effect sizes of these regional differences in the E/I ratio aligned well with the sensorimotor-association (S-A) axis of cortical organization (66), such that effect sizes were lowest at the sensorimotor pole and largest at the association pole (Fig. 5*F*). Spearman’s correlation between effect sizes and S-A axis ranks was *r* = 0.87 (two-tailed spin test *P* < 0.001; Fig. 5*G*). Overall, these results suggest that a more mature E/I ratio—especially in higher-order association cortex—is linked to more mature cognition.

To evaluate the robustness of these results, we repeated these analyses 5 times with different random training-validation splits of participants within each high-performance group and each low-performance group. The results were similar across the 5 splits (*SI Appendix*, Figs. S20–S24; the most representative split is displayed in Fig. 5). Within the most representative split, we found that relaxing the thresholds to between 2.5 Hz and 3.5 Hz yielded similar results (*SI Appendix*, Fig. S25) as did the use of a 100-region homotopic functional parcellation (64) (*SI Appendix*, Fig. S26). Pairwise comparisons between the control analyses are found in *SI Appendix*, Fig. S27.

### 1.7. Results Generalize to a Younger Asian Cohort.

**Fig. 6.**
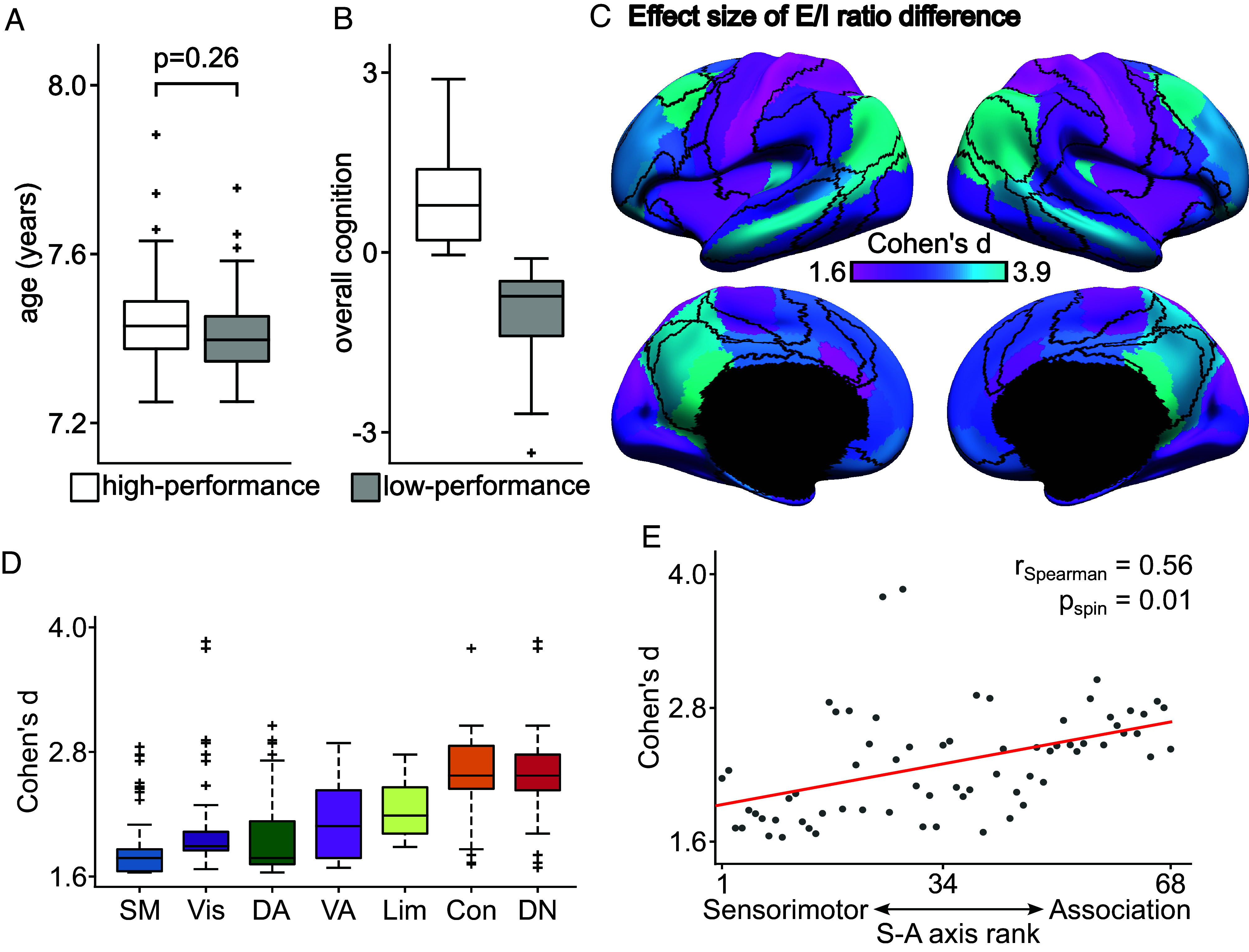
Lower E/I ratio is associated with better cognitive performance within the same age group in the GUSTO dataset. (*A*) Boxplots of age for high-performance and low-performance groups. (*B*) Overall cognition of high-performance and low-performance groups. (*C*) Spatial distribution of effect size of E/I ratio difference between low-performance group and high-performance group. (*D*) Effect size of E/I ratio differences is larger in control and default networks compared with sensory-motor regions. The boxes show the interquartile range (IQR) and the median. Whiskers indicate 1.5 IQR. Black crosses represent outliers. (*E*) Agreement between effect size of E/I ratio difference and S-A axis rank. Spearman’s correlation *r* = 0.56, two-tailed spin test *P* = 0.01.

As a final step, we evaluated whether the link between the E/I ratio and cognition generalized to a group of younger participants of different ancestry. This was motivated by recent concerns that relationships between resting-fMRI and behavior may not generalize well across ethnic groups (67). We utilized the GUSTO dataset (47), which included 154 participants (after quality control) with a mean age of 7.5 y. An overall cognitive performance score was obtained by a principal component analysis of five cognitive tests. Participants were then divided into groups with high and low cognitive performance. The ages were well-matched between the high- and low-performance groups (Fig. 6 *A* and *B*). There was no motion difference between the high- and low-performance groups during the fMRI scans (*P* > 0.1).

Replicating PNC results, we found that the high-performance group exhibited a lower E/I ratio in higher-order association cortex than the low-performance group (Fig. 6*C*). Differences were largest in the default and control networks (Fig. 6*D*). Statistical significance was evaluated using a permutation test, where the null distribution was constructed by randomly assigning participants into high or low-performance groups and then re-estimating the E/I ratio. We note that only 29 (out of 68) regions were significant after FDR correction with *q* < 0.05. These 29 regions were all in association cortex. By contrast, differences in the E/I ratio between cognitive performance groups were largely not significant in sensory-motor networks. As in the PNC, we found the effect sizes of these cognitive differences aligned with the S–A axis (*r* = 0.56, two-tailed spin test *P* = 0.01; Fig. 6*E*). These results in a younger Asian cohort emphasize the robustness and generalizability of our findings.

## 2. Discussion

We first established that the pFIC model could generate realistic fMRI dynamics in a large adult dataset. We then demonstrated that our E/I ratio marker was sensitive to increased inhibitory activity induced by benzodiazepine alprazolam. In a large developmental sample from North America, we found that the E/I ratio marker decreased with age. We also demonstrated that a lower E/I ratio marker—reflective of more mature cortex—was associated with better cognitive performance, particularly in the transmodal association cortex. Critically, these findings generalized to a younger Asian cohort. Together, our findings provide evidence that refinements in the cortical E/I ratio persist into adolescence, suggesting that prolonged E/I-linked developmental plasticity in the association cortex supports continued neurocognitive development. We speculate that insufficient refinement of the E/I ratio during development may create a vulnerability to cognitive deficits, with potentially important implications for transdiagnostic psychopathology.

The E/I ratio is challenging to be noninvasively investigated in humans. Post-mortem studies have established how the expressions of E/I relevant genes vary across the cortex (35, 68, 69). On the other hand, there is a lack of a direct mapping of in vivo neuroimaging signals with excitatory and inhibitory neurobiology, as well as constrained spatial coverage and specificity of available E/I techniques (24–26). Here, we capitalized on recent developments in biologically interpretable computational modeling of cortical circuits to gain insight into the E/I ratio from fMRI data. We fitted a large-scale circuit model with interacting excitatory and inhibitory populations to resting-state fMRI and calculated the E/I ratio from the time courses of excitatory and inhibitory synaptic gating variables. Our E/I ratio marker captured reductions in the E/I ratio induced by alprazolam, a positive allosteric modulator that increases the effectiveness of GABAergic signaling (70). Furthermore, the spatial pattern of benzodiazepine-related E/I reductions described by the model was correlated with the distribution of benzodiazepine-sensitive GABA receptors from positron emission tomography (63). Interestingly, one of the pharmacological targets of benzodiazepines is GABA_A_ α_1_ receptors (70). Increases in GABA signaling at GABA_A_ α_1_ receptors have been shown to trigger the onset of developmental critical periods in animal models (71), indicating that the pFIC model is well equipped to study development-linked changes in inhibitory signaling in the human brain.

We found that the E/I ratio decreased across the cortex throughout child and adolescent development. E/I ratio declined with age across all cortical systems, but the magnitude of decline varied along a unimodal-transmodal cortical hierarchy. Specifically, by age 22, the E/I ratio had declined the most in unimodal sensory territories, such as the visual and somatomotor systems, and the least in transmodal systems like the default and frontoparietal control systems. Differences in E/I development across these systems may be linked to differences in their maturational time courses. The development of cortical inhibitory circuitry is well-established as a central mechanism that controls the timing and progression of critical periods of development (21, 72). Initially, the development of inhibitory circuitry lags behind that of excitatory pyramidal cells, leading to an early increase in the local E/I ratio (73). Later, experience and evoked activity stimulate the development of inhibitory circuitry (21)—particularly fast-spiking parvalbumin-positive interneurons and GABA_A_ α_1_ receptors—which begins to reduce the E/I ratio, facilitating experience-dependent plasticity and triggering the opening of the critical period window (22, 71, 72). As the critical period progresses, excitatory synapses are pruned, further reducing the E/I ratio (74, 75). Finally, as inhibitory circuitry reaches maturity, a different set of plasticity braking factors are triggered, including the formation of intracortical myelin and perineuronal nets, which stabilize cortical circuits and close the critical period window (6, 76). Consequently, an initial decrease in the E/I ratio can signify that a critical period has been triggered and the cortex is in a relatively immature, plasticity-permissive state. As the E/I ratio reduces further, it may signify that the cortex has reached a mature, plasticity-restrictive state, with pruned excitatory synapses, fully developed inhibitory circuitry, and mature plasticity brakes that have closed the critical period (6). As such, the greater reduction in the E/I ratio we observe in sensory systems relative to association systems may reflect that sensory systems have reached a higher degree of maturity by the end of the adolescent years while association systems remain in a more immature, plasticity-permissive state. To test this hypothesis, future work could use multimodal neuroimaging that combines our pFIC approach with other markers of critical period closure—such as intracortical myelination—to evaluate biologically relevant signatures of when windows of critical period plasticity open and close during youth.

Our findings align with a wealth of literature demonstrating differences in the development of sensorimotor and association cortices. Studies have shown that FC, functional topography, structure–function coupling, and intrinsic dynamics follow different developmental trajectories between sensory and associative cortical systems (16, 77–79). For example, while the intrinsic fluctuation amplitude of sensory systems linearly decline with age, association systems follow curvilinear developmental trajectories that peak over adolescence before declining into adulthood (80). Importantly, other recent work has shown that the development of intracortical myelin, which functions as a brake on plasticity, also varies along the sensory-to-association axis (11, 81). Specifically, the period of peak growth in intracortical myelin occurs during childhood in the sensorimotor cortex, yet not until adolescence in the association cortex. Coupled with our current findings of a greater reduction in the E/I ratio of sensory systems (versus a weaker reduction in association systems), this work jointly indicates that sensorimotor systems are more mature by the onset of adolescence, whereas the association cortex may remain more plastic during the adolescent period. This interpretation aligns with a recent study showing that an fMRI marker of functional plasticity peaks during early adolescence in the association cortex but continuously declines throughout childhood and adolescence in the sensorimotor cortex (80).

The protracted development of the E/I ratio throughout adolescence may facilitate healthy cognitive development. We found that better cognitive ability was associated with a lower E/I ratio across the cortex in groups of age-matched youth. Since the E/I ratio normatively decreased with age, this effect may indicate that more mature cognitive performance is associated with a more mature cortical E/I ratio. As such, the E/I ratio may capture the aspects of development independent of chronological age. Importantly, the magnitude of the effect was not spatially uniform. The greatest effect sizes were observed in association cortex, while the weakest effect sizes were observed in sensory cortex. This pattern is consistent with prior work showing that functional properties of the association cortex are most strongly related to cognitive performance across development (79, 82). Our findings also support theoretical predictions from a biophysically based cortical circuit model of decision-making that a balanced E/I ratio supports optimal decision-making (9). Together, our results suggest that although E/I ratio continues to develop throughout the cortex during adolescence, the development of the E/I ratio in the association cortex is particularly relevant to maturing cognition. Critically, we generalized associations between the E/I ratio and cognitive ability in an independent sample of youth collected from a different continent, demonstrating the robustness of these effects across both populations and recruitment sites.

Our findings have important implications for understanding the emergence of psychopathology during adolescence. Though a prolonged period of developmental plasticity in the association cortex may be essential to healthy cognitive development, it may also represent a period of vulnerability to atypical developmental outcomes. A growing body of work has begun to implicate a disrupted E/I ratio in the prefrontal cortex as a central mechanism in neuropsychiatric disorders such as depression and psychosis spectrum disorders (83–85).

These conditions are thought to involve an atypically high E/I ratio in the prefrontal cortex (86–89). Future studies can use our model to understand how the atypical development of E/I ratio in association cortex may lead to transdiagnostic cognitive dysfunction in developmental psychopathology.

### 2.1. Limitations and Future Work.

Parameterization of local circuit parameters with the T1w/T2w ratio map and the FC gradient yielded more realistic fMRI dynamics than either gradient alone or if local circuit parameters were constrained to be spatially uniform. Future studies can explore more generic parameterizations, such as geometric eigenmodes (90).

The current study utilized parcellations with only 68 or 100 regions. Simulating the FIC model with a higher spatial resolution is computationally challenging because the number of inter-regional connections increases quadratically with the number of regions. Future work can explore more efficient algorithms. Furthermore, our analyses were limited to linear modeling of E/I ratio across a set of age bins. Future work in larger samples may facilitate the estimation of nonlinear developmental trajectories of E/I ratio.

Finally, our approach can also be used to study E/I ratio changes during cognitive tasks or during a naturalistic paradigm. When applying the pFIC model to a different dataset, dataset-specific SC, T1w/T2w ratio map and FC gradient can be used, although that might not be necessary. For example, SC, T1w/T2w ratio map, and whole-cortex FC gradient were not available in the alprazolam dataset, so we utilized SC, T1w/T2w, and whole-cortex FC gradient from the HCP dataset.

### 2.2. Conclusion.

Our results underscore the utility of large-scale circuit models to provide insights into the mechanisms driving neurocognitive development. We find that an essential aspect of healthy brain function—the cortical E/I ratio—is refined during childhood and adolescence. We also provide evidence that this hallmark critical period mechanism is associated with improved cognitive ability. Our findings pave the way for future work to investigate how disrupted E/I balance may lead to cognitive dysfunction in psychopathology that emerges during youth and is characterized by atypical development of the association cortex that undergoes protracted maturation.

## 3. Methods

We utilized the HCP S1200 release (N = 1,004; Fig. 2), pharmacological (benzodiazepine alprazolam) fMRI dataset (N = 45; Fig. 3), PNC dataset (N = 885; Figs. 4 and 5) and the GUSTO cohort (N = 154; Fig. 6). In the case of HCP, we used the publicly available ICA-FIX MSMAll resting-state fMRI data in fsLR surface space. For alprazolam and PNC datasets, we used preprocessed fMRI data from our previous study, which involved slice time correction, motion correction, field distortion correction, and anatomical CompCor (26). In the case of the alprazolam dataset, no resting-state fMRI was available, so we used task-fMRI after regressing out the task regressors, following our previous study (26). To be consistent, the GUSTO dataset was also preprocessed in a similar fashion as the PNC dataset. More details can be found in *SI Appendix*, *Supplementary Methods*.

After preprocessing, static FC was computed using Pearson’s correlation for all datasets. FCD was computed using a sliding window length of ~60 s, corresponding to windows of length 83, 20, 20, and 23 for the HCP, alprazolam, PNC, and GUSTO datasets, respectively. The window length followed best practice recommendations from previous studies (51, 91). SC was computed based on the number of streamlines generated with probabilistic tractography using MRtrix3 (92). More details can be found in *SI Appendix*, *Supplementary Methods*.

The pFIC model (33) was fitted to the different datasets using the CMA-ES (56). The fitted pFIC model was used to simulate the synaptic gating variable time courses S_E_ and S_I_ of the excitatory and inhibitory populations, respectively. The E/I ratio was defined as the ratio between the temporal average of S_E_ and S_I_. More details can be found in *SI Appendix*, *Supplementary Methods*.

The HCP data collection was approved by a consortium of institutional review boards (IRBs) in the United States and Europe, led by Washington University in St Louis and the University of Minnesota (WU-Minn HCP Consortium). Data collection and study procedures for the Alprazolam dataset were approved by the University of Pennsylvania IRB; data collection for the PNC was approved by IRBs from both the University of Pennsylvania and the Children’s Hospital of Philadelphia. The GUSTO data collection was approved by the National Healthcare Group Domain Specific Review Board and the SingHealth Centralised Institutional Review Board. All participants provided informed consent before data collection. The current study was approved by the IRB of the National University of Singapore.

## Supplementary Material

Appendix 01 (PDF)

## Data Availability

The HCP data are publicly available (https://www.humanconnectome.org) (93). The GUSTO dataset can be obtained via a data transfer agreement (www.gusto.sg) (94). The PNC dataset is publicly available in the Database of Genotypes and Phenotypes (https://www.ncbi.nlm.nih.gov/projects/gap/cgi-bin/study.cgi?study_id=phs000607.v3.p2) (95). All pharmacological imaging data necessary to evaluate the conclusions in the paper are available here (https://github.com/ThomasYeoLab/CBIG/tree/master/stable_projects/fMRI_dynamics/Zhang2024_pFIC/replication/Alprazolam) (96). Code for this study can be found here (https://github.com/ThomasYeoLab/CBIG/tree/master/stable_projects/fMRI_dynamics/Zhang2024_pFIC) (97). Coauthors (T.Z. and L.A.) reviewed the code before merging into the GitHub repository to reduce the chance of coding errors. Previously published data were used for this work (43–45, 47).
