# Supplementary material for: In vivo whole-cortex marker of excitation-inhibition ratio indexes cortical maturation and cognitive ability in youth
Source: Proc Natl Acad Sci U S A. 2024 May 30;121(23):e2318641121. doi: 10.1073/pnas.2318641121 (PMC11161789; doi:10.1073/pnas.2318641121)
Supplement: Supplementary file 1 — Appendix 01 (PDF) [file pnas.2318641121.sapp.pdf]

In vivo whole-cortex marker of excitation-inhibition ratio indexes cortical maturation and cognitive ability in youth

Supplemental Material

This supplemental material consists of Supplemental Methods and Supplemental Results to complement the Methods and Results sections in the main text.

Supplemental Methods

S1. Human Connectome Project (HCP) dataset

We considered 1004 participants from the Human Connectome Project (HCP) S1200 release (1). All participants were scanned on a customized Siemens 3T Skyra using a multi-band sequence. Four resting-state fMRI (resting-fMRI) runs were collected for each participant in two sessions on two different days. Each resting-fMRI run was acquired with a repetition time (TR) of 0.72 s at 2 mm isotropic resolution and lasted for 14.4 min. The diffusion imaging consisted of 6 runs, each lasting ~9 min and 50 s. Diffusion weighting consisted of 3 shells of b = 1000, 2000, and 3000 s/mm^2 with an approximately equal number of weighting directions on each shell. Details of the data collection can be found elsewhere (1). The 1004 participants were randomly divided into training (N = 335), validation (N = 335) and test (N = 334) sets.

S2. HCP preprocessing

Details of the HCP preprocessing can be found in the HCP S1200 manual. We utilized resting-fMRI data, which had already been projected to fsLR surface space, denoised with ICA-FIX and smoothed by 2 mm. For each run of each participant, the fMRI data were averaged within each Desikan–Killiany (2) region of interest (ROI) to generate a 68 × 1200 matrix. Each 68 × 1200 matrix was used to compute 68 × 68 FC matrix by correlating the time courses among all pairs of time courses. The FC matrices were then averaged across runs of participants within the training (or validation or test) set, resulting in a group-averaged training (or validation or test) FC matrix.

Functional connectivity dynamics (FCD) were computed as follows. We defined a window with a length of 60s (equivalent to 83 time points or TRs) as recommended by previous studies (3,4). The window was moved from the first frame to the 1118th frame of BOLD time series, resulting in 1118 sliding windows in total. For each run of each participant, FC was computed within each of 1118 sliding windows. Each sliding window FC matrix was then vectorized by only considering the upper triangular entries. The vectorized FCs were correlated with each other generating a 1118 × 1118 FCD matrix. Unlike static FC, we note that the FCD matrices could not be directly averaged across participants because there was no temporal correspondence between participants during the resting-state (See section S10).

In the case of diffusion MRI, probabilistic tractography was run for each participant using the fiber orientation distribution (iFOD2) algorithm provided by MRtrix3 (5). A structural connectivity (SC) matrix was generated for each participant, where each entry corresponded to the number of streamlines between two ROIs. To generate a group-level SC matrix, a thresholding procedure was employed to remove false positives. More specifically, if <50% of participants had a non-zero value in a particular entry in the SC matrix, then the

entry is set to zero in all individual-level SC matrices. For each SC entry, the number of streamlines was averaged across participants with non-zero streamlines and then log-transformed (6). The entries along the main diagonal were set to 0. Group-level SCs were computed by averaging individual-level SCs within the training, validation and test sets separately and normalizing the maximum value to 0.02.

S3. Pharmacological (benzodiazepine alprazolam) dataset

The alprazolam dataset has been previously described in detail (7). Briefly, 47 adults participated in a double-blind, placebo-controlled study using the benzodiazepine alprazolam. Each participant completed two identical experimental sessions approximately 1 week apart. In one session, participants were given a 1-mg dose of alprazolam, and in the other, they were given an identical appearing placebo. One milligram of alprazolam produces an increase in GABAergic inhibition that is considered to be clinically effective (8). The order of administration was counterbalanced across participants. Alprazolam or placebo was administered 1 hour before the fMRI acquisition so that alprazolam levels and effects were near their peak at the time of data collection (8). During both sessions, participants completed an emotion identification task lasting 10.5 min, while fMRI was acquired. Task-related fMRI results have been previously reported (7). Two participants were excluded because of missing data in at least one session, yielding a final sample of 45 participants and 90 sessions in total (ages 20.9 to 56.4; mean = 39.9, standard deviation = 12.71).

All data were collected on a Siemens Trio 3T. Blood oxygen level–dependent (BOLD) fMRI data were acquired using the following parameters: TR = 3000 ms; TE = 32 ms; flip angle = 90°; FOV = 240 mm; matrix = 128 × 128; slice thickness/gap = 2/0 mm; 30 slices; effective voxel resolution = 1.875 × 1.875 × 2 mm^3; 210 volumes. The field of view (FOV) included temporal, inferior frontal, and visual cortices as well as subcortical structures, but excluded dorsal portions of the cerebral cortex.

The 45 participants were randomly divided into training, validation, and test sets with 15 participants each. The training-validation-test split was the same for both drug and placebo sessions.

S4. Philadelphia Neurodevelopment Cohort (PNC) dataset

Neuroimaging data were obtained from a community-based sample of 1601 youth (ages 8.1 to 23.1; mean = 14.94; standard deviation = 3.69; male/ female = 764/837) that were part of the Philadelphia Neurodevelopmental Cohort (PNC). Data collection procedures and sample characteristics have been previously described in detail (9,10). One run of resting-fMRI data was collected per participant. Following health exclusions and rigorous quality assurance, we retained 885 participants (ages 8.2 to 23.0 at first visit; mean = 15.66; standard deviation = 3.36).

All neuroimaging data were collected on the same Siemens Trio 3T scanner as was used for the alprazolam dataset. The neuroimaging procedures and acquisition parameters have been previously described in detail (9). Briefly, BOLD fMRI was acquired using similar acquisition parameters to the alprazolam dataset: TR = 3000 ms; TE = 32 ms; flip angle = 90°; FOV = 192 × 192 mm^2; matrix = 64 × 64; 46 slices; slice thickness/gap = 3/0 mm; effective voxel resolution = 3.0 × 3.0 × 3.0 mm^3; 210 volumes. The key difference is that the field of view in the PNC dataset covered the whole brain (unlike the alprazolam dataset).

S5. Alprazolam and PNC functional image processing

Details about the alprazolam and PNC datasets have been previously provided in previous studies (11). The alprazolam dataset consisted of two BOLD acquisitions per participant (drug and placebo session), which were preprocessed individually. BOLD runs were slice time–corrected and then motion-corrected. Susceptibility distortion was estimated and used to compute a corrected BOLD reference for more accurate co-registration with the anatomical reference. The BOLD reference was co-registered to the T1w reference using boundary-based registration (12). Co-registration was configured with nine degrees of freedom to account for distortions remaining in the BOLD reference. Six head motion parameters (corresponding rotation and translation parameters) were estimated before any spatiotemporal filtering. The motion-correcting transformations, field distortion correcting warp, BOLD-to-T1w transformation, and T1w-to-template (MNI) warp were concatenated and applied to the BOLD time series in a single step using antsApplyTransforms (ANTs) with Lanczos interpolation. Finally, the volumetric data was projected to fsaverage6 surface space (13).

Nuisance regression relied upon anatomical CompCor (aCompCor). aCompCor principal components were estimated after high-pass filtering the preprocessed BOLD time series (using a discrete cosine filter with 128-s cutoff). 5 CompCor components were extracted from the cerebrospinal fluid (CSF) and white matter (WM) masks. To remove task effects in the alprazolam dataset, all event conditions from the emotion identification task were modeled as 5.5-s boxcars convolved with a canonical hemodynamic response function. Each of the five emotions (fear, sad, angry, happy, and neutral) was modeled as a separate regressor. In sum, 22 regressors (6 head motion parameters and their respective temporal derivatives, top 5 aCompCor components, and 5 task regressors) were jointly regressed from the BOLD time series. The same preprocessing was performed for the PNC dataset, except that no task regressor was necessary. Overall, 17 regressors (6 head motion parameters and their respective temporal derivatives and top 5 aCompCor components) were jointly regressed from the BOLD time series.

FC and FCD of the alprazolam and PNC datasets were computed in the same manner as the HCP dataset. However, the TR was longer in the alprazolam and PNC datasets than the HCP dataset. Therefore, when computing the FCD matrices, the length of each sliding window was set to be 20 timepoints (or TRs), so that the temporal length of the window was maintained at 60s.

S6. GUSTO dataset

To generalize our findings on the association between cognition and E/I ratio, we additionally utilized an Asian cohort, Growing Up in Singapore Towards Healthy Outcomes (GUSTO) dataset (14). We considered 389 7.5-year-old children with 1 run of resting-fMRI data and relevant cognitive scores. Participants with one or more missing cognitive scores were removed, yielding a final group of 154 participants (mean age = 7.43, std = 0.13, min age = 7.24, max age = 7.88).

All neuroimaging data were collected on a Siemens Prisma scanner. The structural data were obtained with T1w MPRAGE sequence using the following acquisition parameters: TR = 2000 ms; TE = 2.08 ms; FOV = 192×192 mm^2; matrix = 192×192; voxel resolution = $1.0 \times 1.0 \times 1.0$ mm^3. The resting-fMRI data were obtained with the following acquisition parameters: TR = 2620 ms; TE = 27 ms; flip angle = 90°; FOV = 192×192 mm^2; matrix =

64 × 64; 48 slices; voxel resolution = 3.0 × 3.0 × 3.0 mm^3; 120 volumes. The field of view of the GUSTO dataset covered the whole brain.

S7. GUSTO preprocessing

For each resting-fMRI run, the following sequence of preprocessing steps were performed. The first 4 frames of the run were removed. The run was then slice time-corrected and then motion-corrected. Frames with FD > 0.5mm and DVARS > 80 were marked as motion outliers. Next, the run was co-registered to the structural image with boundary-based registration (12). Nuisance regression was performed with the inclusion of white matter and CSF signals, 6 head motion parameters and their respective temporal derivates as well as the top 5 aCompCor components (19 regressors in total were jointly applied). The motion outlier frames were then censored and interpolated. Finally, the run was bandpass-filtered (0.009Hz < f < 0.08Hz) and projected to Freesurfer fsaverage6 surface space.

FC and FCD of the GUSTO dataset were computed in the same manner as the HCP dataset. However, the TR was longer in the GUSTO than the HCP dataset. Therefore, when computing the FCD matrices, the length of each sliding window was set to be 23 timepoints (or TRs), so that the temporal length of the window was maintained at around 60s.

S8. Feedback Inhibition Control (FIC) model

The derivation of the FIC model was thoroughly described in a previous study (15). Here we provide some intuition for the FIC model. The neuronal activities of the j-th cortical region follow the nonlinear differential equations shown below

$$I_j^{(E)} = W_E I_0 + w_{EE} J_{NMDA} S_j^{(E)} + G J_{NMDA} \sum_k C_{jk} S_k^{(E)} - w_{IE} S_j^{(I)} \tag{1}$$

$$I_j^{(I)} = W_I I_0 + w_{EI} J_{NMDA} S_j^{(E)} - w_{II} S_j^{(I)} \tag{2}$$

$$r_j^{(E)} = \phi\left(I_j^{(E)}\right) = \frac{a_E I_j^{(E)} - b_E}{1 - \exp\left(-d_E\left(a_E I_j^{(E)} - b_E\right)\right)} \tag{3}$$

$$r_j^{(I)} = \phi\left(I_j^{(I)}\right) = \frac{a_I I_j^{(I)} - b_I}{1 - \exp\left(-d_I\left(a_I I_j^{(I)} - b_I\right)\right)} \tag{4}$$

$$\frac{dS_j^{(E)}}{dt} = -\frac{S_j^{(E)}}{\tau_E} + \left(1 - S_j^{(E)}\right)\gamma r_j^{(E)} + \sigma \nu_j(t) \tag{5}$$

$$\frac{dS_j^{(I)}}{dt} = -\frac{S_j^{(I)}}{\tau_I} + r_j^{(I)} + \sigma \nu_j(t) \tag{6}$$

where S, r, and I represent synaptic gating variables, firing rate, and synaptic currents respectively. The superscripts E and I denote the excitatory and inhibitory neuronal populations respectively.

The input current $I_j^{(E)}$ of the excitatory population of the j-th cortical ROI is the sum of four inputs (Equation 1). The first input is the external input current $W_E I_0$, which might include subcortical delays. The second input is the intra-regional excitatory-to-excitatory current governed by the excitatory-to-excitatory recurrent connection strength w_{EE} scaled by the synaptic coupling constant J_{NMDA}. The third input is the inter-regional input, which is controlled by the SC matrix (C_{jk} is the connectivity between regions j and k) and scaled by the global constant G. The fourth input is the intra-regional negative feedback from the inhibitory population governed by the inhibitory-to-excitatory connection strength w_{IE}.

The input current $I_j^{(I)}$ of the inhibitory population of the j-th cortical ROI is the sum of three inputs (Equation 2). The first input is the external input current $W_I I_0$. The second input is the intra-regional positive feedback from the excitatory population governed by the excitatory-to-inhibitory connection strength w_{EI} scaled by the synaptic coupling constant J_{NMDA}. The third input is the intra-regional inhibitory-to-inhibitory current governed by the inhibitory-to-inhibitory recurrent connection strength w_{II}.

The excitatory input current $I_j^{(E)}$ and inhibitory input current $I_j^{(I)}$ are transformed into firing rates via the input-output functions specified in Equations 3 and 4. Following previous studies (15), the parameters of the input-output function were set to be $a_E = 310$n/C, $a_I = 615$n/C, $b_E = 125$Hz, $b_I = 177$Hz, $d_E = 0.16$s and $d_I = 0.087$s. Finally, the rate of change of the synaptic gating variables $S_j^{(E)}$ and $S_j^{(I)}$ are computed via equations 5 and 6. Following previous studies (15), the kinetic parameters for synaptic activities τ_E, τ_I and γ were set to 100ms, 10ms and 0.641 respectively. $\nu_j(t)$ corresponds to uncorrelated standard Gaussian noise with the noise amplitude being controlled by σ.

Following the original study (15), w_{II}, W_E, W_I, I_0 and J_{NMDA} were set to 1, 1, 0.7 0.382nA and 0.15nA respectively in the current study. The inhibitory-to-excitatory connection strength $w_{IE}$was computed analytically to ensure that the excitatory firing rate is maintained to be around 3Hz (16). We note that this analytical computation assumes a noiseless system, so in practice, we imposed a constraint that the firing rate is between 2.7Hz to 3.3Hz. During the estimation of the pFIC model (next section), parameters were rejected if firing rates fall outside this range.

The excitatory-to-excitatory recurrent connection strength w_{EE}, excitatory-to-inhibitory connection strength w_{EI}, noise amplitude σ and global SC scaling constant G are unknown parameters, which will be estimated by fitting to empirical fMRI data (next section). Given a fixed set of model parameters, equations 1 to 6 can be used to simulate the time courses of excitatory and inhibitory synaptic gating variables ($S_j^{(E)}$ and $S_j^{(I)}$) of each ROI. The regional E/I ratio was defined as the ratio between the temporal average of $S_j^{(E)}$ and $S_j^{(I)}$. The mean cortical E/I ratio was the average of regional E/I ratios across all cortical ROIs. The simulated excitatory synaptic gating variables ($S_j^{(E)}$) were also fed to the Balloon-Windkessel hemodynamic model to simulate fMRI BOLD signals (15, 17,18). The simulated fMRI BOLD signals were then used to generate simulated static FC and FCD.

S9. Parametric FIC (pFIC) model

Recall that the FIC model was instantiated using the Desikan-Killiany parcellation with 68 regions of interest. Given that we wanted the excitatory-to-excitatory recurrent connection strength w_{EE}, excitatory-to-inhibitory connection strength w_{EI} and noise amplitude σ to be spatially heterogeneous (with G being a global constant), if we optimized each parameter independently, there would be a total of 68 × 3 + 1 = 205 parameters, which is computationally challenging.

In our previous study (19), we reduced the number of "free" parameters by parameterizing the local synaptic parameters with a linear combination of the first principal FC gradient and T1w/T2w ratio map. We note that in the previous study (19), we considered a highly simplified parametric mean field model that does not differentiate between excitatory and inhibitory neural populations. Here, we considered the same approach for the FIC model by parameterizing w_{EE}, w_{EI}, and σ as a linear combination of T1w/T2w myelin map and first principal FC gradient.

$$w_{EE,j} = a + b \times \text{myelin}_j + c \times \text{FC gradient}_j \tag{7}$$

$$w_{EI,j} = d + e \times \text{myelin}_j + f \times \text{FC gradient}_j \tag{8}$$

$$\sigma_j = g + h \times \text{myelin}_j + i \times \text{FC gradient}_j \tag{9}$$

where j denotes the ROI index. By adopting this parameterization approach, the number of "free" numbers was reduced to 3 × 3 + 1 = 10 parameters.

S10. Optimization of the parametric FIC (pFIC) model in the HCP dataset

The 10 unknown parameters of the pFIC model were optimized using a previously published approach (19) by maximizing fit to empirical static FC and FCD. The agreement between empirical and simulated FC matrices was defined as the Pearson's correlation (r) between the z-transformed upper triangular entries of the two matrices. Larger r indicates more similar static FC. However, Pearson's correlation does not account for scale difference, so we also computed the absolute difference (d) between the means of the empirical and simulated FC matrices (16). A smaller d indicates more similar static FC.

We note that there is no temporal correspondence between simulated and empirical FCD matrices, so we cannot simply use the Euclidean distance to measure dissimilarity. Instead, the dissimilarity between simulated and empirical FCD matrices was quantified by using the Kolmogorov-Smirnov (KS) distance. Here, the KS distance was defined as the maximum distance between the cumulative distribution functions (CDFs) constructed by collapsing the upper triangular entries of simulated and empirical FCD matrices (19,20). Hence, a small KS distance indicated 2 similar CDFs, therefore 2 similar FCD matrices. Because the KS distance was computed by collapsing the upper triangular entries of the FCD matrices, no temporal correspondence was assumed.

The overall cost was defined as $(1 – r) + d + KS$. A smaller cost indicates better agreement between simulated and empirical fMRI. Recall that the 1004 HCP participants were randomly divided into training (N = 335), validation (N = 335) and test (N = 334) sets. Following our previous study (19), we used the covariance matrix adaptation evolution strategy (CMA-ES) (21) to minimize the overall cost function during training. Due to the lack

of temporal correspondence between FCD matrices across runs and participants during rs-fMRI scans, directly averaging FCD matrices will cancel out the temporal dynamics. Representing FCD matrices using CDFs avoided such problem because averaging the CDFs across runs and participants could still largely preserve the distribution of FCD matrix entries. We thus averaged the FCD CDFs across participants and runs separately within the training, validation and test sets.

In the HCP training set, the CMA-ES algorithm was iterated 100 times and repeated 5 times with different random initializations, yielding a total of 500 candidate parameter sets (**Figure 2A**). The 500 candidate parameter sets were evaluated in the validation set to obtain the top 10 candidate parameter sets. To ensure diversity among the parameter sets, the procedure to select the top 10 parameter sets was as follows. First, the parameter set with the lowest validation cost was selected. Then, the parameter set with the lowest validation cost and whose parameter (w_{EE}, w_{EI}, σ) maps exhibited less than 0.98 correlation with the current selected parameter set(s) was selected. This procedure was repeated until 10 parameter sets were selected.

The top 10 candidate parameter sets from CMA-ES were then applied to the HCP test set SC. For each parameter set, 1000 simulations were performed, yielding 1000 simulated static FC and FCD matrices. Pearson correlation and the absolute difference were then computed between each simulated FC and the empirical FC from the HCP test set and averaged. Similarly, KS statistics was computed between each simulated FCD CDF and the empirical FCD CDF from the HCP test set and averaged.

To speed up the computation, a step size of 6ms was used to integrate the ODEs in the training set. However, to ensure more accurate integration, a step size of 0.5ms was used for both validation and test sets. To ensure that this time step size was small enough, we repeated the experiment using a step size of 6ms for the training set and a step size of 0.1ms for the validation and test sets. The overall cost was highly similar across step size of 0.5ms and step size of 0.1ms. In particular, for both 0.5ms and 0.1ms step sizes, an overall cost of 0.58 ± 0.018 was achieved in the HCP test set across the top 10 parameter sets from the validation set.

Additionally, we observed that the correlations between w_{EI} and T1w/T2w ratio were consistently negative, while the correlations between w_{EI} and RSFC gradient were consistently positive across the top 10 parameter sets. Since the training sets for the alprazolam and PNC datasets were substantially smaller than the HCP training set (~15 participants versus 335 participants), when optimizing the pFIC model in the alprazolam, PNC and GUSTO datasets, we additionally imposed the constraints that w_{EI} and T1w/T2w ratio should be negative, while the correlations between w_{EI} and FC gradient should be positive.

It is worth noting that when evaluating the top 10 model estimates (selected from the HCP validation set) in the HCP test set, the correlation loss (1-*r*) ranged from 0.27 to 0.29, absolute difference loss *d* ranged from 0.08 to 0.14, and the *KS* distance ranged from 0.14 to 0.23. In our previous study (19), our cost function was (1-*r*) + *KS*. Changing the relative weights of (1-*r*) and *KS* did not substantially change the model estimate. Therefore, we kept the weights unchanged in the current study. However, we observed that the simulated fMRI time courses were overly synchronized, so we included the additional absolute difference (*d*) metric in the current study. We observed that setting the relative weight of *d* to be the same as the other 2 terms was sufficient to prevent the simulated fMRI time courses from becoming

over-synchronized (**Figure S1**). Therefore, we did not consider changing the relative weights further.

S11. Pharmacological E/I ratio analysis

Recall that the 45 participants of the alprazolam dataset were randomly divided into training (N = 15), validation (N = 15) and test (N = 15) sets. Each participant had 2 runs of fMRI data - one run for the drug session and one run for the placebo session. Because there was no diffusion data in the alprazolam dataset, the SC matrices used for training, validation, and testing were the same as HCP training, validation, and tests set respectively. The T1w/T2w ratio map also had to be generated from HCP training set.

Due to the limited FOV of the alprazolam dataset, we only considered regions with more than 50% ROI coverage, resulting in 42 ROIs (in the case of the Desikan-Killiany parcellation). The remaining 26 ROIs were masked out for the group-level SC, static FC, FCD, T1w/T2w ratio. Furthermore, given the limited FOV, the first principal FC gradient was generated from HCP training set and the 26 ROIs were masked out similarly. For each experimental condition (placebo or alprazolam), 250 candidate parameter sets were generated from the condition's training set. The top 10 parameter sets from the validation set were evaluated in the test set.

Although the parameters were only estimated based on the 42 ROIs, the estimated linear coefficients could be used to generate whole cortex estimates of w_{EE}, w_{EI} and σ (based on equations 7 to 9) given that the original T1w/T2w ratio and FC gradient (from the HCP training set) covered the whole cortex. The simulated time courses S_E and S_I were then generated using the 68-ROI SC and extrapolated model parameters. For a given set of parameters, 1000 simulations were performed to generate 1000 sets of E/I ratio. The final E/I ratio was the average across 1000 sets of E/I ratio.

E/I ratio contrast was defined as the difference between E/I ratio of the placebo sessions and E/I ratio of the drug sessions. To test that the E/I ratio contrast was significantly greater than 0, we performed permutation test to generate a null distribution of E/I ratio contrasts. More specifically, after dividing the participants into training, validation and test sets, the 'drug' and 'placebo' sessions were randomly permuted within each participant. The entire procedure (above) was repeated, generating a null value for the E/I ratio contrast. The permutation procedure was repeated 100 times, yielding a null distribution of regional E/I ratio contrasts. A 2-tail p-value was computed based on this null distribution.

The regional E/I ratio contrast was also correlated with benzodiazepine receptor (BZR) density. The statistical significance of this correspondence was computed using a spin test that accounts for spatial autocorrelation (22).

S12. Association between age and E/I ratio in the PNC dataset

885 participants of the PNC dataset (9) were first sorted according to age in ascending order and divided into 29 groups of 30 or 31 participants. For each age group, 15 participants were randomly selected as the validation set, while the remaining participants were assigned to the training set.

To be consistent with previous analyses, the SC matrices used for training and validation were the same as HCP training and validation sets respectively. Both T1w/T2w ratio and the first principal FC gradient maps were generated from HCP training set, consistent with the alprazolam analyses. For each age group, 250 candidate model parameter

sets were generated from the group's training set using CMA-ES and evaluated in the group's validation set. For each age group, the parameter set with the lowest validation cost was used to estimate regional E/I ratio across the cortex.

For each cortical ROI, we fitted a linear regression model between regional E/I ratio and the mean age of each age group. The slope of the linear regression model (for each brain region) was visualized on the cortical surface. All slopes were negative and all p-values survived FDR correction (q < 0.05). For robustness, the split of the participants into training and validation sets were repeated 5 times and the most representative split was shown in **Figure 4**.

S13. Association between cognition and E/I ratio in the PNC dataset

Each PNC participant had completed a set of 12 tasks from 4 cognitive domains, including executive control, episodic memory, complex cognition, and social cognition. Three types of scores, including an accuracy score, a speed score, and an efficiency score, were obtained for each of the 12 tasks. Factor analyses of each type of scores were performed within each cognitive domain and across all cognitive domains to generate three domain-specific accuracy factor scores and one domain-general (overall) accuracy score (23).

To control for age, 885 participants were sorted according to age in an ascending order. For each 12-month interval, participants whose age were within this interval were extracted to form one age group. For each age group, participants with domain-general (overall) accuracy scores above the median were assigned to a high-performance group, the rest are assigned to a low-performance group. In sum, 441 participants were assigned to the high-performance group (mean age = 15.68), 444 participants were assigned to the low-performance group (mean age = 15.64). Both high and low-performance groups were then divided into 14 subgroups of 31 (or 32) participants, yielding 14 pairs of age-matched high-performance and low-performance groups (see **Figure 5A and 5B**).

Participants of each subgroup were further randomly divided into a training set (N = 16) and a validation set (N = 15 or 16). Similar to the previous analyses, SCs used for training and validation were from the HCP training and validation sets respectively. T1w/T2w ratio and FC gradient maps were from the HCP training set. The E/I ratios of the 14 pairs of high-performance and low-performance groups were estimated separately and compared using a 2-tail 1-sample t-test. To test for domain specificity, we also repeated the analyses for the 3 domain-specific accuracy scores. FDR correction with $q < 0.05$ was used to correct for multiple comparisons.

S14. Association between cognition and E/I ratio in the GUSTO dataset

Our analyses were based on the fMRI and behavioral data of 154 participants from the GUSTO dataset. The fMRI data were acquired when the participants were around 7.5 years old. We selected 5 behavioral scores which assessed participants' cognitive performances. All behavior tests were performed within 1.5 years of fMRI acquisition (i.e., age 6 to 8.5). The 5 test scores were as follows: (1) Cambridge Neuropsychological Test Automated Battery (CANTAB) Spatial Working Memory (SWM) test (completed at age 6): sum of total errors for 4 and 6 boxes trails. (2) Delayed Matching to Sample (DMS) test (completed at age 6): percentage of the total number of trials upon which a correct selection was made on the participant's first response. (3) Behavior Rating Inventory of Executive Function (BRIEF; completed at age 7): Cognition Regulation Index T-score. (4) Wechsler

Abbreviated Scale of Intelligence (WASI) test (completed at age 7): sum of Block Design and Matrix Reasoning T-scores. (5) CANTAB SWM test (completed at age 8.5): sum of total errors for 4 and 8 boxes trails.

Principal component analysis (PCA) was performed on these 5 scores across all participants to derive the first principal component (PC1) score. Higher PC1 score indicated better cognitive performance across the 5 behavioral scores (on average). Participants were sorted according to their PC1 scores in an ascending order. The first 77 participants were assigned to the low-performance group, while the rest of 77 participants were assigned to the high-performance group. Ages were well-matched between high and low-performance groups (**Figure 6A**).

Participants of high and low-performance groups were further randomly divided into a training set (N = 39) and a validation set (N = 38). Similar to the previous analyses, SCs used for training and validation were from the HCP training and validation sets respectively. T1w/T2w ratio and FC gradient maps were from the HCP training set. For robustness, the analyses were repeated 5 times with different random training-validation splits of participants within high-performance group and each low-performance group. Results from the most representative split were shown in the results.

To compute the statistical significance of E/I ratio differences between low and high-performance groups, PC1 scores were randomly permuted across participants. The participants were again assigned to high or low-performance groups according to their permuted PC1 scores. Then the E/I ratio difference was re-estimated. This permutation process was repeated 100 times to construct a null distribution of E/I ratio difference. A 2-tail p-value was computed based on this null distribution.

References

1. Van Essen, D. C. *et al.* The WU-Minn Human Connectome Project: An overview. *NeuroImage* **80**, 62–79 (2013).

2. Desikan, R. S. *et al.* An automated labeling system for subdividing the human cerebral cortex on MRI scans into gyral based regions of interest. *NeuroImage* **31**, 968–980 (2006).

3. Leonardi, N. & Van De Ville, D. On spurious and real fluctuations of dynamic functional connectivity during rest. *NeuroImage* **104**, 430–436 (2015).

4. Liégeois, R., Laumann, T. O., Snyder, A. Z., Zhou, J. & Yeo, B. T. T. Interpreting temporal fluctuations in resting-state functional connectivity MRI. *NeuroImage* **163**, 437–455 (2017).

5. Tournier, J.-D. *et al.* MRtrix3: A fast, flexible and open software framework for medical image processing and visualisation. *NeuroImage* **202**, 116137 (2019).

6. Park, B. *et al.* Differences in subcortico-cortical interactions identified from connectome and microcircuit models in autism. *Nat Commun* **12**, 2225 (2021).

7. Wolf, D. H. *et al.* Amygdala abnormalities in first-degree relatives of individuals with schizophrenia unmasked by benzodiazepine challenge. *Psychopharmacology* **218**, 503–512 (2011).

8. Greenblatt, D. J., Harmatz, J. S., Dorsey, C. & Shader, R. I. Comparative single-dose kinetics and dynamics of lorazepam, alprazolam, prazepam, and placebo. *Clin Pharmacol Ther* **44**, 326–334 (1988).

9. Satterthwaite, T. D. *et al.* Neuroimaging of the Philadelphia Neurodevelopmental Cohort. *NeuroImage* **86**, 544–553 (2014).

10. Calkins, M. E. *et al.* The Philadelphia Neurodevelopmental Cohort: constructing a deep phenotyping collaborative. *J Child Psychol Psychiatr* **56**, 1356–1369 (2015).

11. Larsen, B. *et al.* A developmental reduction of the excitation:inhibition ratio in association cortex during adolescence. *Sci. Adv.* **8**, eabj8750 (2022).

12. Greve, D. N. & Fischl, B. Accurate and robust brain image alignment using boundary-based registration. *NeuroImage* **48**, 63–72 (2009).

13. Wu, J. *et al.* Accurate nonlinear mapping between MNI volumetric and FreeSurfer surface coordinate systems. *Hum. Brain Mapp.* **39**, 3793–3808 (2018).

14. Soh, S.-E. *et al.* Cohort Profile: Growing Up in Singapore Towards healthy Outcomes (GUSTO) birth cohort study. *International Journal of Epidemiology* **43**, 1401–1409 (2014).

15. Deco, G. *et al.* How Local Excitation-Inhibition Ratio Impacts the Whole Brain Dynamics. *Journal of Neuroscience* **34**, 7886–7898 (2014).

16. Demirtaş, M. *et al.* Hierarchical Heterogeneity across Human Cortex Shapes Large-Scale Neural Dynamics. *Neuron* **101**, 1181-1194.e13 (2019).

17. Stephan, K. E., Weiskopf, N., Drysdale, P. M., Robinson, P. A. & Friston, K. J. Comparing hemodynamic models with DCM. *NeuroImage* **38**, 387–401 (2007).

18. Heinzle, J., Koopmans, P. J., Den Ouden, H. E. M., Raman, S. & Stephan, K. E. A hemodynamic model for layered BOLD signals. *NeuroImage* **125**, 556–570 (2016).

19. Kong, X. *et al.* Sensory-motor cortices shape functional connectivity dynamics in the human brain. *Nat Commun* **12**, 6373 (2021).

20. Hansen, E. C. A., Battaglia, D., Spiegler, A., Deco, G. & Jirsa, V. K. Functional connectivity dynamics: Modeling the switching behavior of the resting state. *NeuroImage* **105**, 525–535 (2015).

21. Hansen, N. The CMA Evolution Strategy: A Comparing Review. in *Towards a New Evolutionary Computation* (eds. Lozano, J. A., Larrañaga, P., Inza, I. & Bengoetxea, E.) vol. 192 75–102 (Springer Berlin Heidelberg, Berlin, Heidelberg, 2006).

22. Alexander-Bloch, A. F. *et al.* On testing for spatial correspondence between maps of human brain structure and function. *NeuroImage* **178**, 540–551 (2018).

23. Moore, T. M., Reise, S. P., Gur, R. E., Hakonarson, H. & Gur, R. C. Psychometric properties of the Penn Computerized Neurocognitive Battery. *Neuropsychology* **29**, 235–246 (2015).

Supplemental Figures

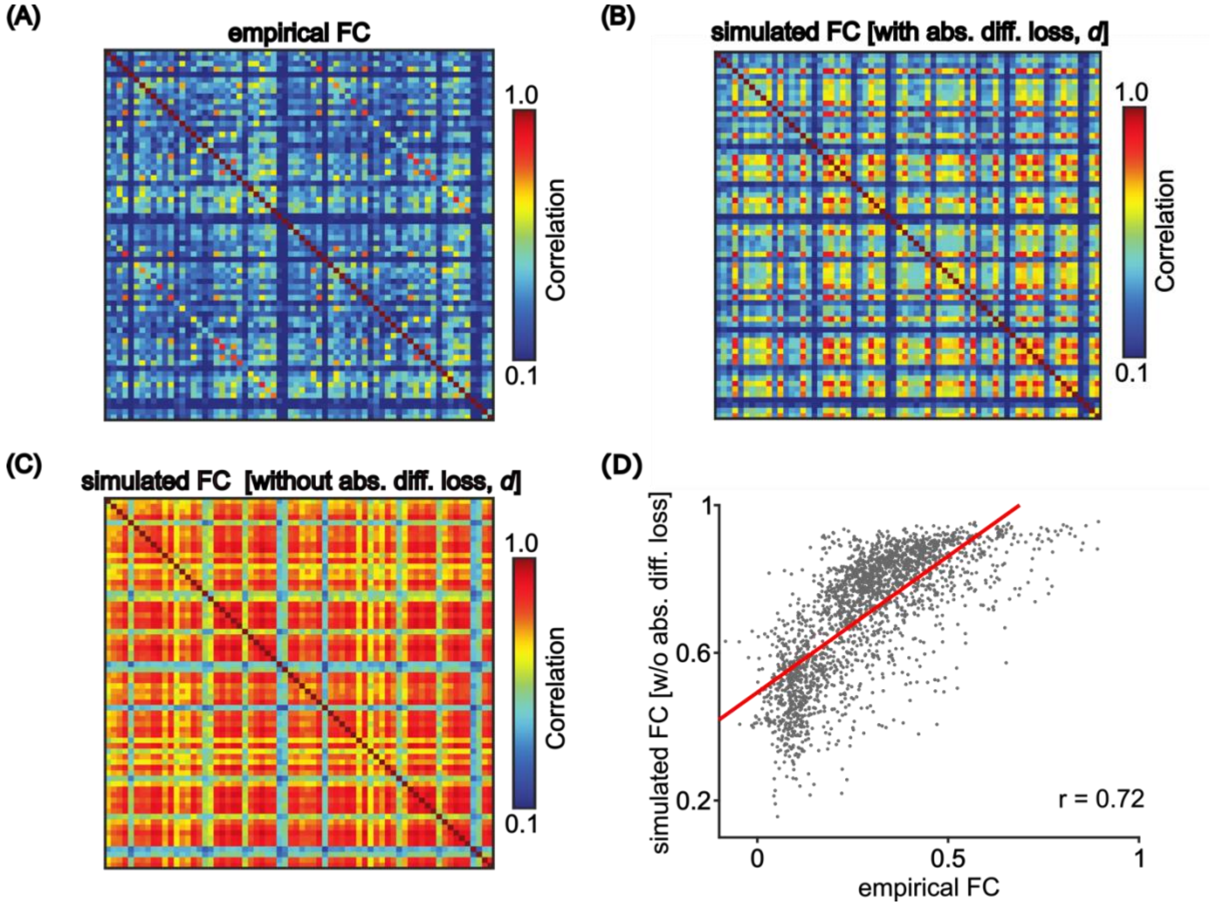

Figure S1. Importance of absolute difference (*d*) metric to prevent overly synchronized simulated fMRI time series. (A) Empirical FC from the HCP test set. (B) Simulated FC from the pFIC model using the best model parameters (from the validation set) and SC from the test set when the cost function contains all three terms: (1 – *r*) + *d* + *KS*. The cost function contained two terms related to static FC: disagreement between empirical and simulated FC in terms of Pearson's correlation (1 – *r*) and absolute difference (*d*). (C) Simulated FC from the pFIC model using the best model parameters (from the validation set) and SC from the test set when the cost function contained only two terms: (1 – *r*) + *KS*. (D) Agreement (Pearson's correlation) between empirical and simulated static FC when the when the cost function contained only two terms: (1 – *r*) + *KS*. Therefore, without the inclusion of the absolute difference (*d*) metric, we can obtain good correlation agreement between simulated and empirical FC. However, by comparing panels (B) and (C), we observe that the lack of the absolute difference (*d*) metric leads to overly synchronized fMRI signals, compared with the empirical FC in panel A. It is also worth noting that the over-synchronization phenomenon was also observed in our previous study (19), which used (1 – *r*) + *KS* as the cost function. Therefore, in this study, we added the absolute difference (*d*) cost to the cost function.

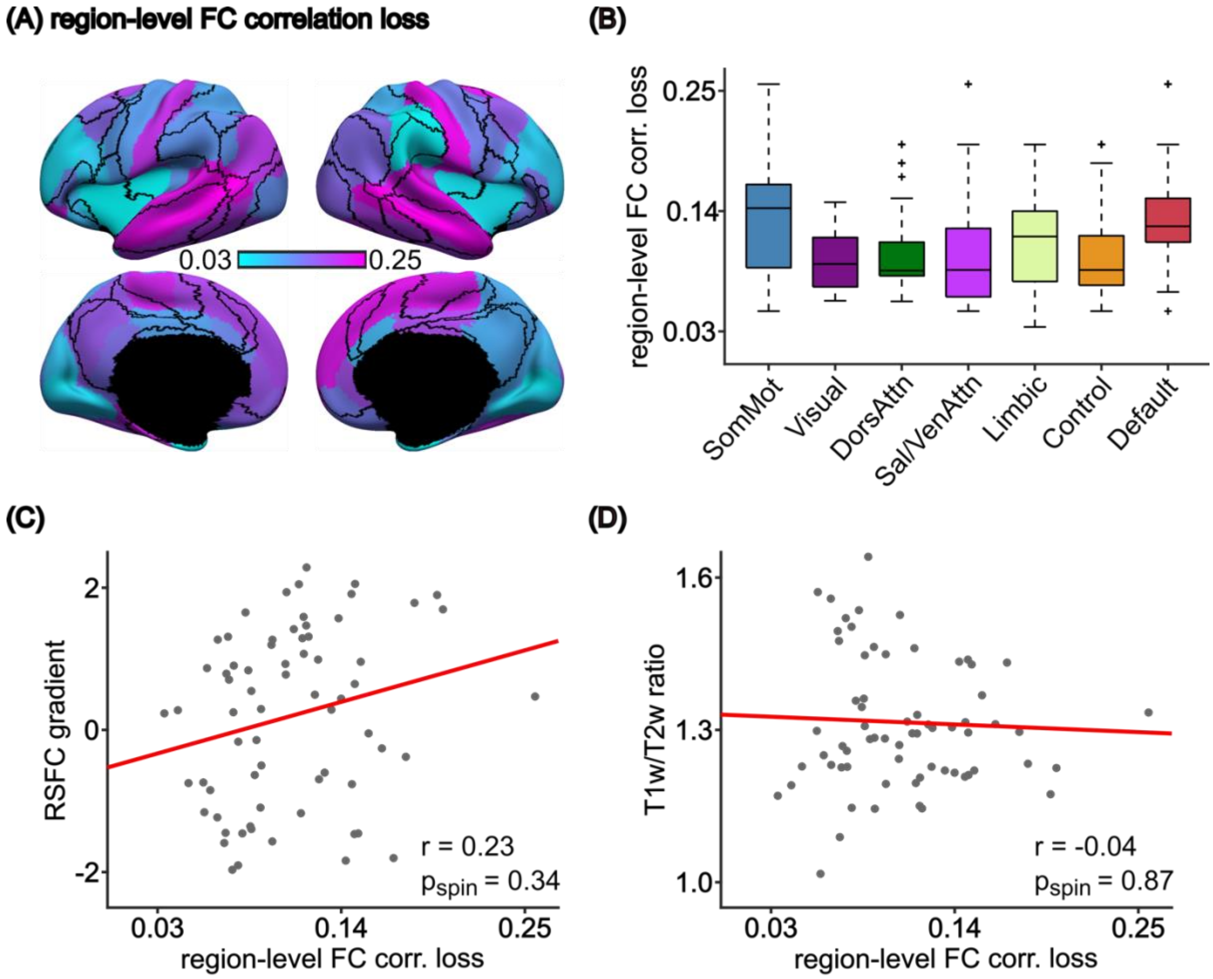

Figure S2. Regional evaluation of pFIC model for static FC. (A) Spatial distribution of region-level FC loss (dissimilarity) between simulated and empirical fMRI in the HCP test set. The regional FC correlation loss was defined as (1 - r_i), where r_i is the Pearson's correlation between the *i*-th rows of empirical and simulated static FC matrices. (B) Regional FC correlation losses grouped by different large-scale networks. The boxes show the inter-quartile range (IQR) and the median. Whiskers indicate 1.5 IQR. Black crosses represent outliers (C) There was no significant correlation between RSFC gradient and the regional FC correlation loss (r = 0.23, two-tail spin test p = 0.34). (D) There was no significant correlation between T1w/T2w ratio and the regional FC correlation loss (r = -0.04, two-tail spin test p = 0.87).

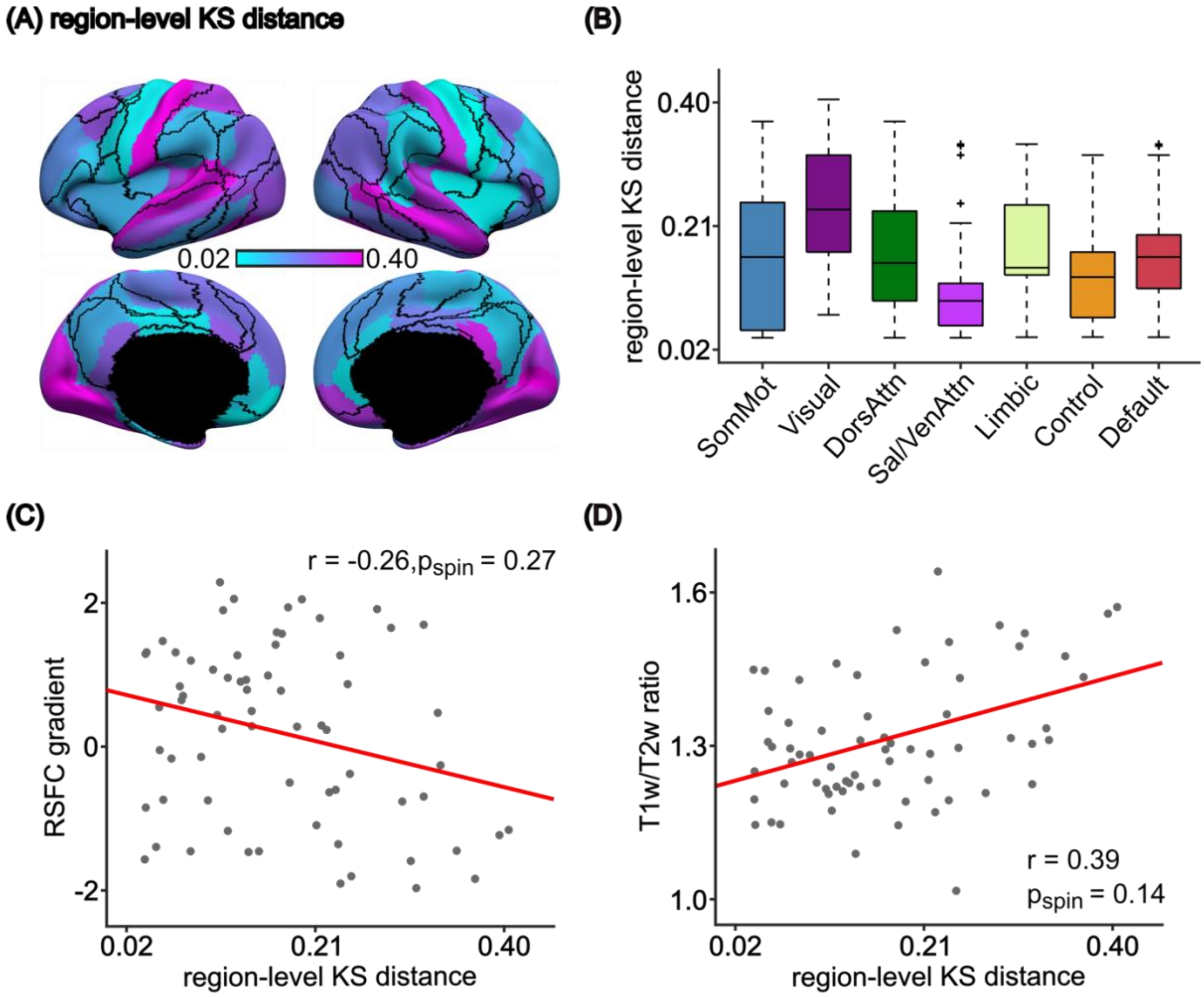

Figure S3. Regional evaluation of pFIC model for FCD. (A) Spatial distribution of region-level FCD dissimilarity (KS distance) between empirical and simulated FCD in the HCP test set. To compute regional KS distance, recall that we have previously computed a 68 × 68 FC matrix for each sliding window (1118 sliding windows in total). For each region, the corresponding rows of the 68 × 68 FC matrices were then correlated across the 1118 windows, yielding a 1118 × 1118 FCD matrix for each region. The KS distance can thus be computed for each region. (B) Regional KS distances grouped by different networks. The boxes show the inter-quartile range (IQR) and the median. Whiskers indicate 1.5 IQR. Black crosses represent outliers. (C) There was no significant correlation between RSFC gradient and regional KS distance (r = -0.26, two-tail spin test p = 0.27). (D) There was no significant correlation between T1w/T2w ratio and regional KS distance (r = 0.39, two-tail spin test p = 0.14).

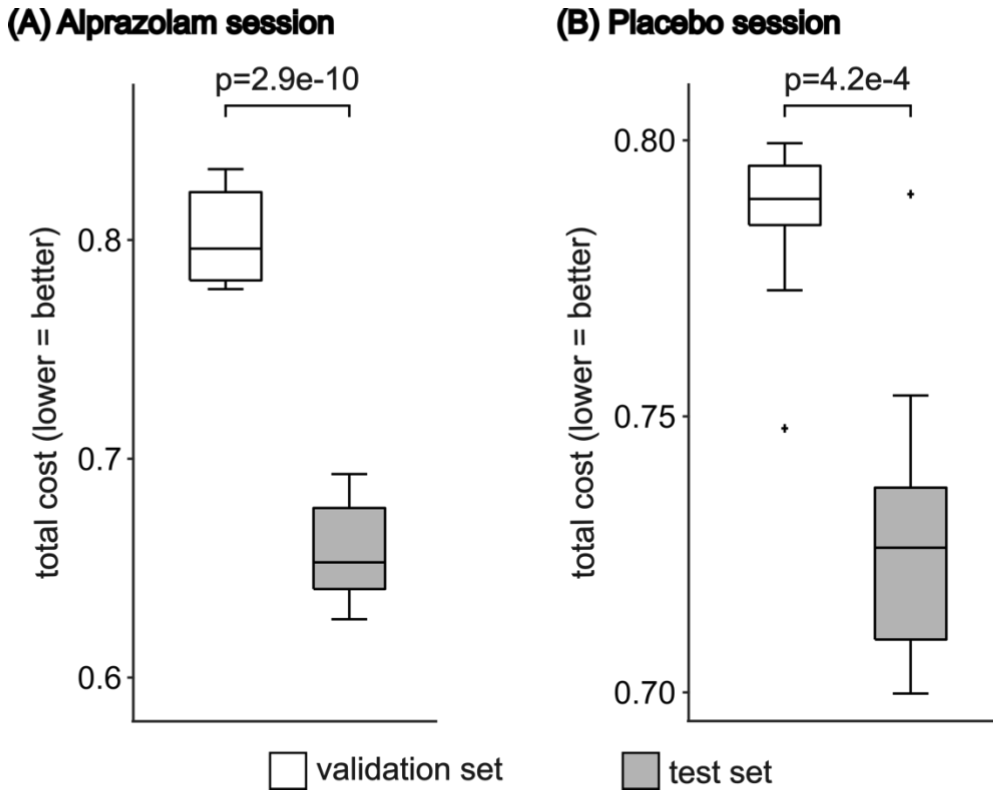

Figure S4. (A) Comparison of the total costs in the validation set and the test set of the alprazolam session. (B) Comparison of the total costs in the validation set and the test set of the placebo session. The total costs in the validation set correspond to the lowest 10 validation costs, generated from the top 10 sets of parameters from the validation set. The total costs of the test set were computed using the same 10 sets of parameters and the structural connectivity of the test set. For both alprazolam and placebo sessions, total costs of the test set were significantly lower than those of the validation set, suggesting that the parameters generalized well from the validation set to the test set. The boxes show the inter-quartile range (IQR) and the median. Whiskers indicate 1.5 IQR. Black crosses represent outliers.

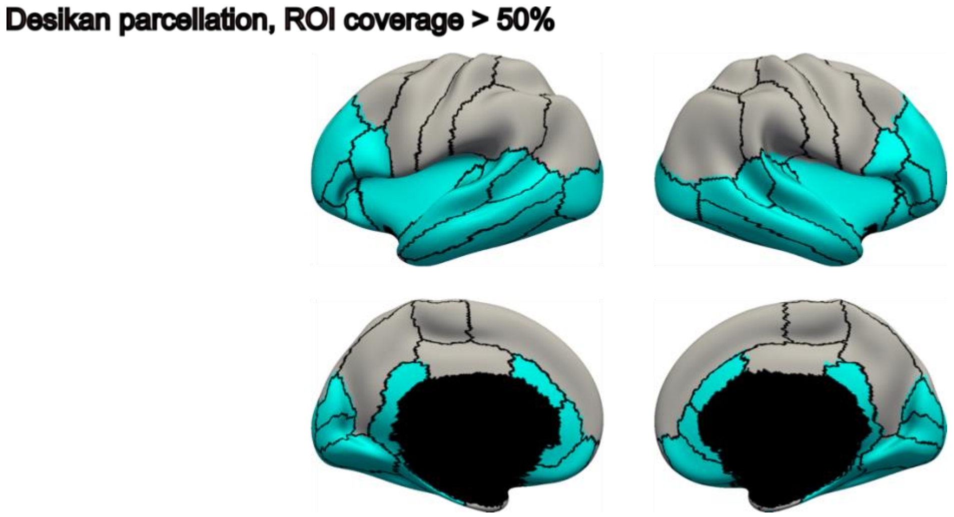

Figure S5. Desikan parcellation with 50% ROI coverage threshold. Due to limited FOV of the alprazolam dataset, only ROIs with coverage higher than a pre-specified threshold were included. ROIs included (excluded) for analysis are colored in cyan (grey). 42 out of 68 ROIs are included.

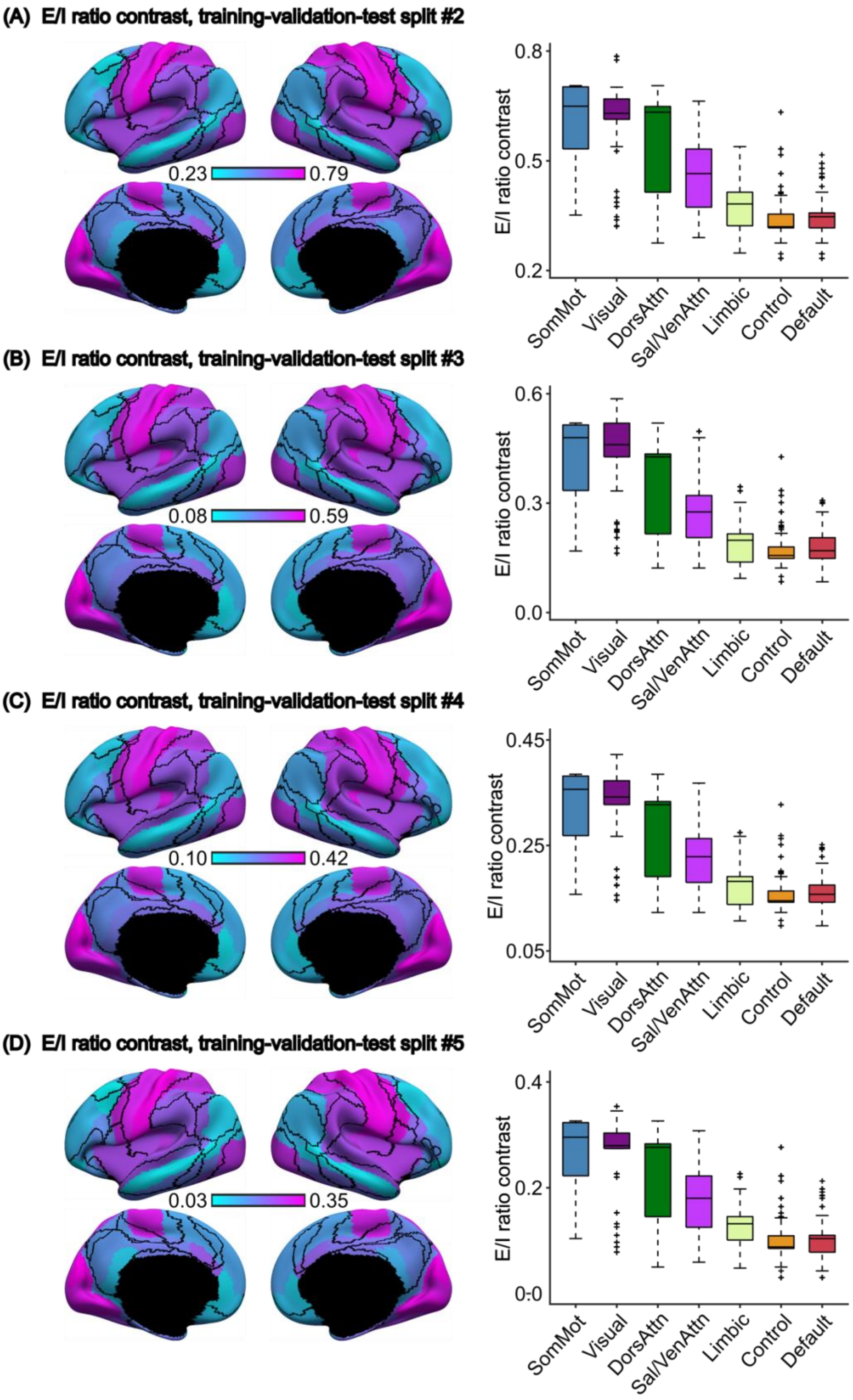

Figure S6. (A to D) As a control analysis of the alprazolam E/I ratio contrast result, the same analysis was replicated with 4 additional training-validation-test participant splits. E/I ratio contrast was defined as the E/I ratio difference between the placebo session and the drug

session. 45 participants were randomly assigned to a different training, validation, and test set. (Left) Spatial distribution of E/I ratio contrast between placebo and drug session. (Right) The E/I ratio contrast decreases along a sensory-to-association axis. The boxes show the inter-quartile range (IQR) and the median. Whiskers indicate 1.5 IQR. Black crosses represent outliers.

	split #1	split #2	split #3	split #4	split #5
split #1	1	0.9936	0.9922	0.9948	0.9853
split #2		1	0.9800	0.9896	0.9949
split #3			1	0.9979	0.9802
split #4				1	0.9909
split #5					1

Figure S7. Pairwise correlation between the regional E/I ratio contrast across different training-validation-test participant splits (split #1-5). The spatial distribution of region E/I ratio contrast are highly similar across splits (r = 0.9899 ± 0.0062, mean ± std). Only the upper triangle of the matrix is shown. We chose the split that had the highest median correlation of regional E/I ratio contrast with the other 4 splits as the most representative split (i.e., split #1) and showed as Figure 3 of the main text.

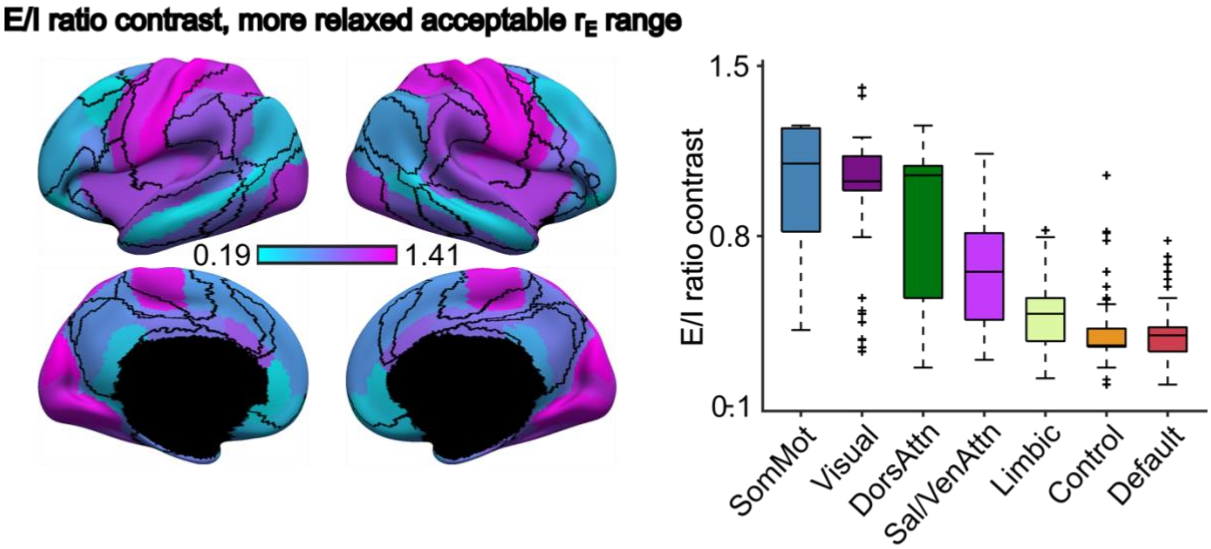

Figure S8. As a control analysis of the alprazolam E/I ratio contrast result, the acceptable excitatory firing rate range was set to be less strict. The range was changed from 2.7 – 3.3Hz to 2.5 – 3.5 Hz. We repeated the same analysis, and the model parameters were retrained using the same optimization approach. The boxes show the inter-quartile range (IQR) and the median. Whiskers indicate 1.5 IQR. Black crosses represent outliers.

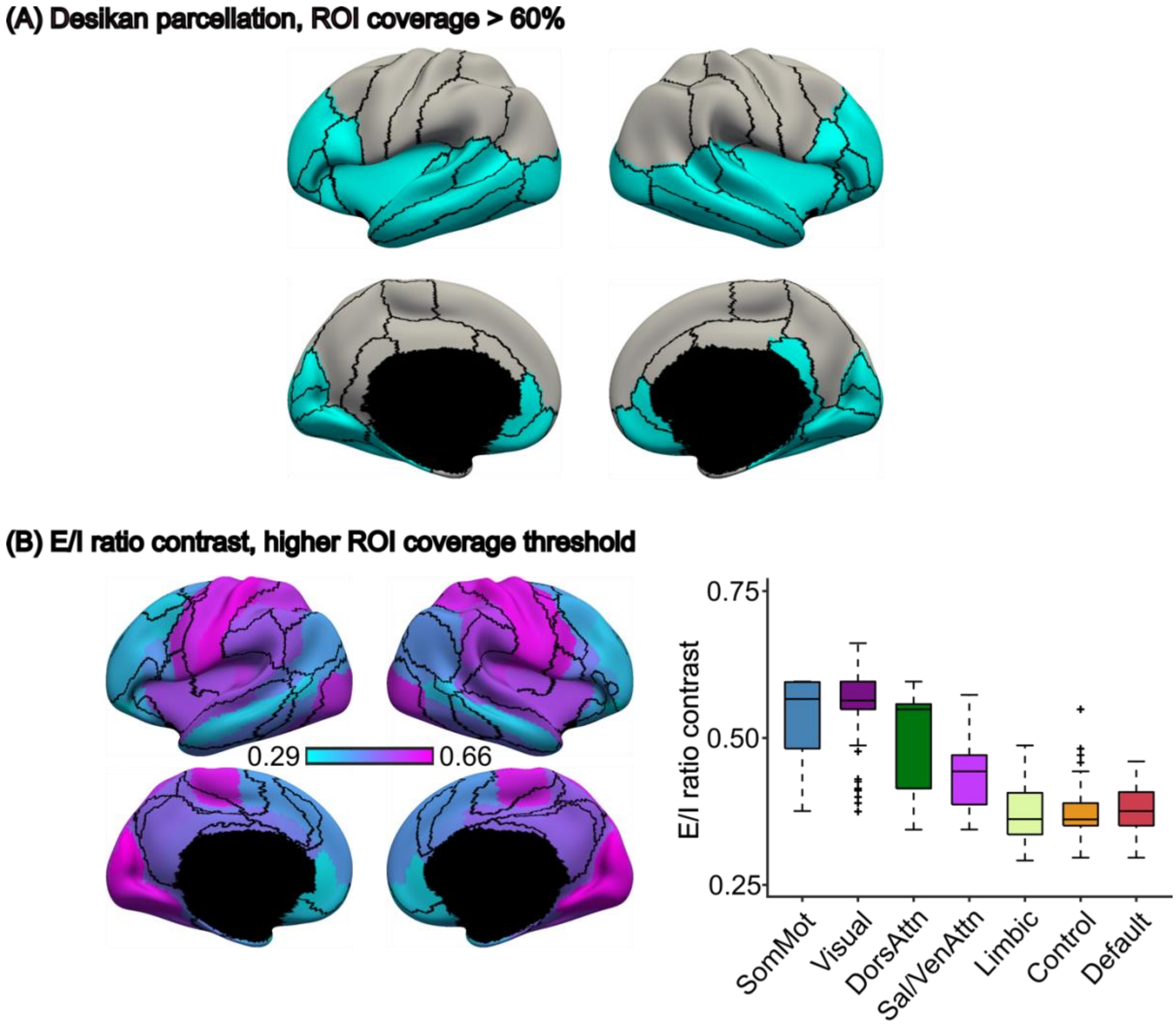

Figure S9. (A) Desikan parcellation with 60% ROI coverage threshold, 39 out of 68 ROIs are left. (B) As a control analysis of the alprazolam E/I ratio contrast result, the ROI coverage threshold was changed to be stricter. The threshold was raised from 50% to 60%. As a result, 3 more ROIs were removed from analysis. We repeated the same analysis, and the model parameters were retrained using the same optimization approach. The boxes show the inter-quartile range (IQR) and the median. Whiskers indicate 1.5 IQR. Black crosses represent outliers.

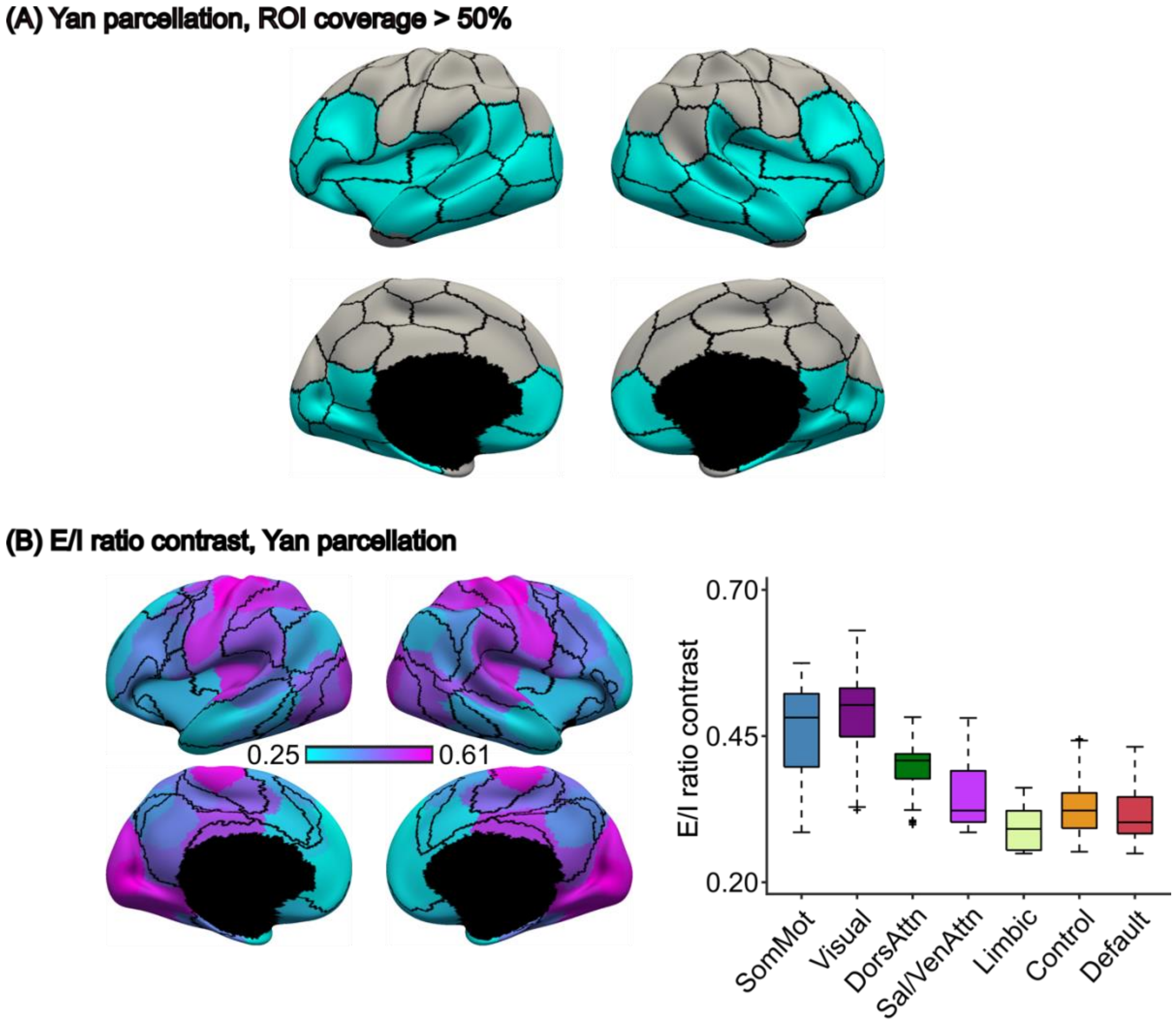

Figure S10. (A) Yan parcellation with 50% ROI coverage threshold, 51 out of 100 ROIs are left. (B) As a control analysis of the alprazolam E/I ratio contrast result, we changed the parcellation scheme to a higher-resolution 100-ROI parcellation. Yan parcellation has ROIs that are symmetric for the left and right hemispheres. We repeated the same analysis, and the model parameters were retrained using the same optimization approach. The boxes show the inter-quartile range (IQR) and the median. Whiskers indicate 1.5 IQR. Black crosses represent outliers.

	split #1	relaxed r_E range	higher ROI coverage threshold	Yan
split #1	1	0.9911	0.9941	0.8986
relaxed r_E range		1	0.9758	0.8422
higher ROI coverage threshold			1	0.9220
Yan				1

Figure S11. Pairwise correlation between the regional E/I ratio contrast across different control analyses based on split #1. Split #1 corresponds to the results shown in Figure 3 of the main text. The spatial distribution of region E/I ratio contrast are highly similar across different control analyses (r = 0.9373 ± 0.0606, mean ± std). Only the upper triangle of the matrix is shown.

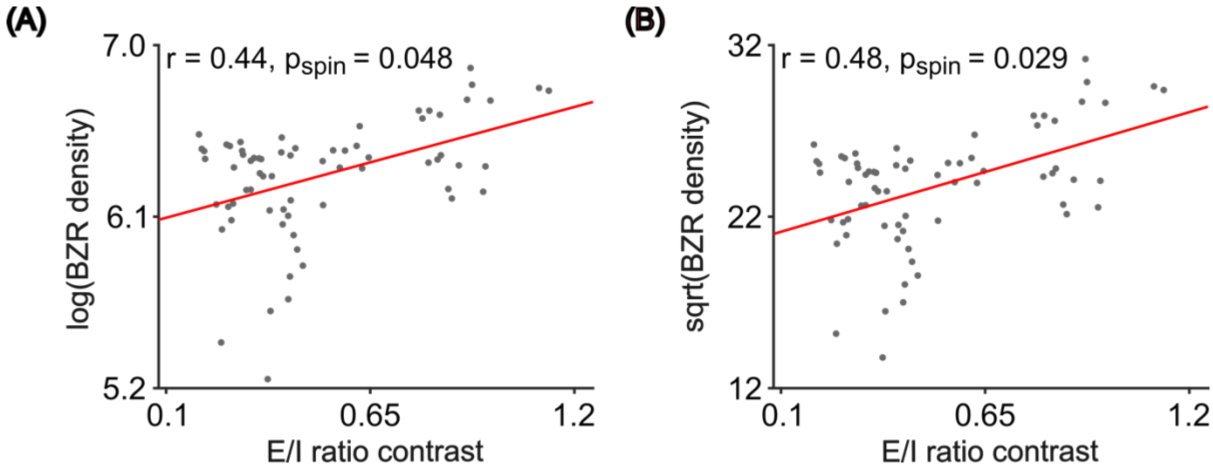

Figure S12. (A) Spatial correlation between regional E/I ratio contrast and log-transformed benzodiazepine receptor (BZR) density (r = 0.44). (B) Spatial correlation between regional E/I ratio contrast and the square root of BZR density (r = 0.48). Both correlations were weaker than the main results (Figure 3D), although the correlations remained statistically significant.

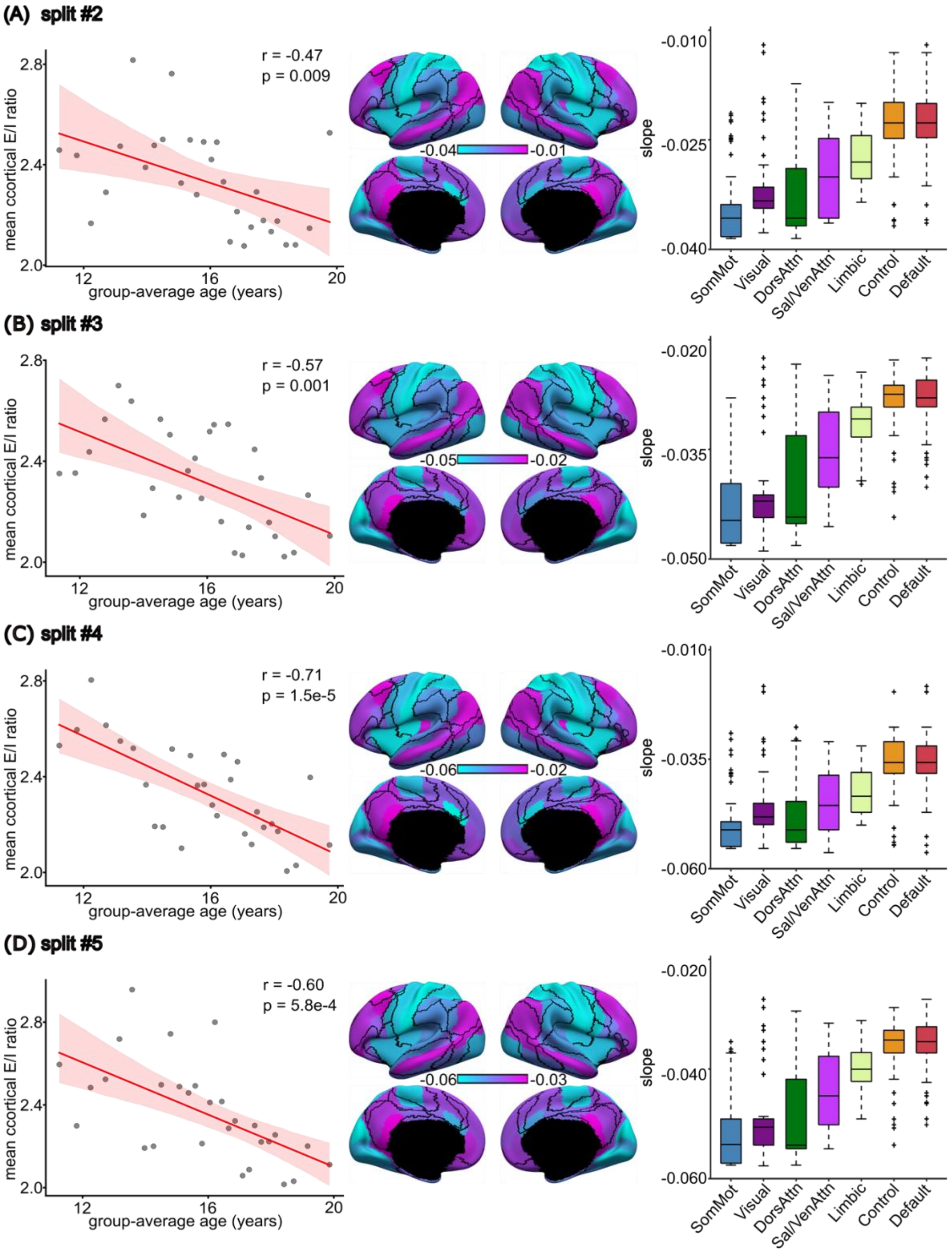

Figure S13. (A to D) PNC developmental analysis results obtained from 4 additional training-validation participants splits. (Left) The mean cortical E/I ratio decreases with increasing age. (Middle) The spatial distribution of regional rate of E/I ratio reduction. (Right) The regional rate of E/I ratio reduction followed a hierarchical sensorimotor-association (S-A) axis. To generate a training-validation split, 885 PNC participants were sorted according to age in an ascending order and divided into 29 age groups. Within each age groups, participants were

randomly assigned to training and validation sets. The random participant splits and analyses were repeated 5 times (One shown in Figure 4, the other four shown here). All 5 splits exhibited the similar pattern of overall E/I ratio reduction and spatial distribution of the rate of reduction. We chose the split that had the highest median correlation of regional rate of E/I ratio reduction with the other 4 splits as the most representative split and showed in the main result section. The shaded area depicts 95% confidence interval of the linear relationship. The boxes show the inter-quartile range (IQR) and the median. Whiskers indicate 1.5 IQR. Black crosses represent outliers.

	split #1	split #2	split #3	split #4	split #5
split #1	1	0.9776	0.9777	0.9253	0.9921
split #2		1	0.9167	0.9835	0.9501
split #3			1	0.8295	0.9954
split #4				1	0.8783
split #5					1

Figure S14. Pairwise correlation between the regional rate of E/I ratio reduction of 5 training-validation participant splits. Split #1 corresponds to the results shown in Figure 4 of the main text. The spatial distributions of the rate of E/I ratio reduction are highly similar across the 5 splits (r = 0.9426 ± 0.0551, mean ± std). The surface maps of different splits are shown in Figure S13. Only the upper triangle of the matrix is shown.

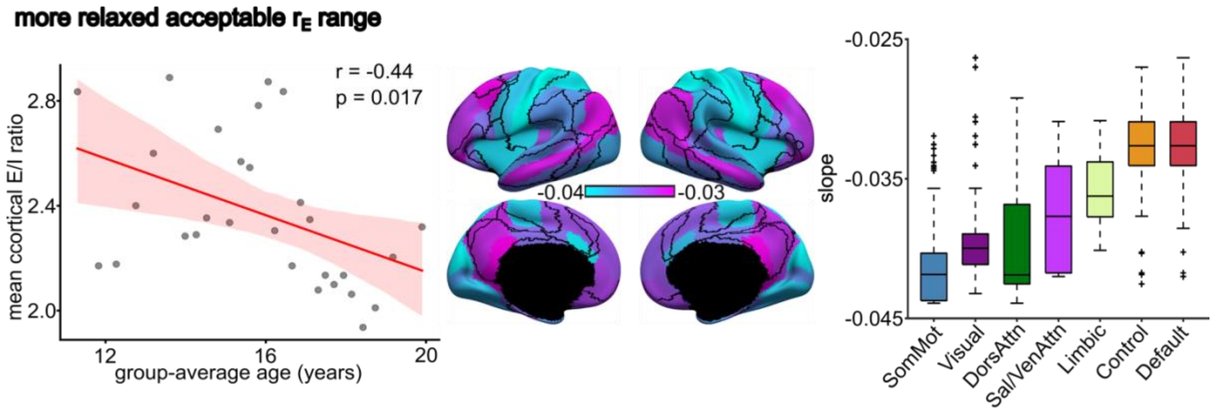

Figure S15. PNC developmental analysis results with relaxed excitatory firing rates thresholds. This figure is similar to Figure 4 but with the acceptable excitatory firing rates range was set to be less strict (2.5Hz to 3.5Hz). (Left) The scatter plot of overall E/I ratio reduction during development. The mean cortical E/I ratio is the average E/I ratio across all ROIs. (Middle) The spatial distribution of region E/I ratio reduction rate. (Right) Box plot of the vertex-level E/I ratio grouped by 7 resting-state networks. The boxplots comprised values obtained by "transferring" the parameter estimates from the 68 Desikan parcels to all vertices (from the underlying cortical meshes) comprising each anatomical parcel. The vertex wise parameter values were then segregated based on the seven resting-state networks. Therefore, there were 3203, 2478, 1523, 1520, 1067, 1438 and 2886 values comprising the boxplots for somatomotor, visual, dorsal attention, ventral attention, limbic, control and default networks respectively. The shaded area depicts 95% confidence interval of the linear relationship. The boxes show the inter-quartile range (IQR) and the median. Whiskers indicate 1.5 IQR. Black crosses represent outliers. The rate of E/I ratio reduction follows the sensorimotor-association (S-A) axis. E/I ratio exhibits the fastest rate of reduction in sensory regions compared to association regions.

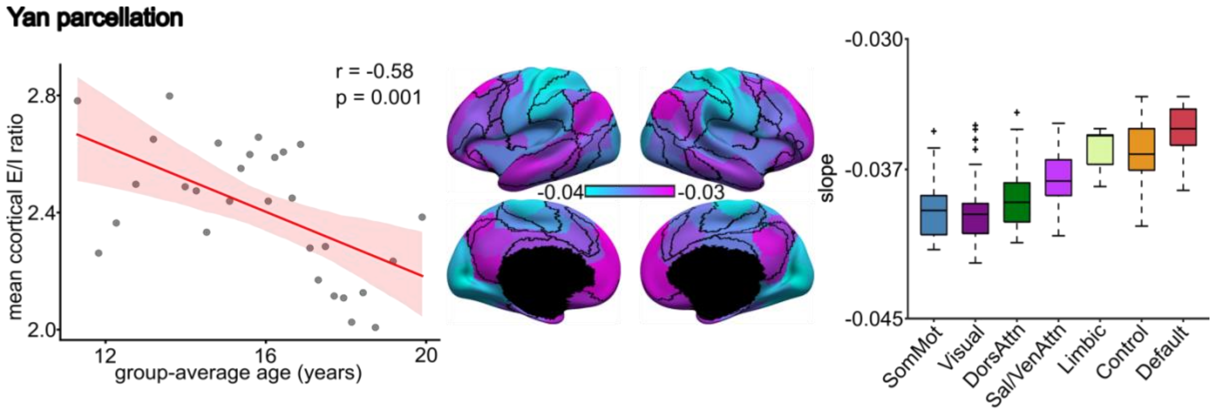

Figure S16. PNC developmental analysis results in Yan 100-ROI parcellation. This figure is similar to Figure 4 but utilizes the Yan 100-ROI parcellation with symmetric left and right hemisphere ROIs. (Left) The scatter plot of overall E/I ratio reduction during development. The mean cortical E/I ratio is the average E/I ratio across all ROIs. (Middle) The spatial distribution of region E/I ratio reduction rate. (Right) Box plot of the vertex-level E/I ratio grouped by 7 resting-state networks. The boxplots comprised values obtained by "transferring" the parameter estimates from the 100 Yan parcels to all vertices (from the underlying cortical meshes) comprising each anatomical parcel. The vertex wise parameter values were then segregated based on the seven resting-state networks. Therefore, there were 3203, 2478, 1523, 1520, 1067, 1438 and 2886 values comprising the boxplots for somatomotor, visual, dorsal attention, ventral attention, limbic, control and default networks respectively. The shaded area depicts 95% confidence interval of the linear relationship. The boxes show the inter-quartile range (IQR) and the median. Whiskers indicate 1.5 IQR. Black crosses represent outliers. The rate of E/I ratio reduction follows the sensorimotor-association (S-A) axis. E/I ratio exhibits the fastest rate of reduction in sensory regions compared to association regions.

	split #1	relaxed r_E range	Yan
split #1	1	0.9887	0.7622
relaxed r_E range		1	0.6791
Yan			1

Figure S17. Pairwise correlation between the regional rate of E/I ratio reduction of different control analyses based on split #1. Split #1 corresponds to the results shown in Figure 4 of the main text. The spatial distributions of the rate of E/I ratio reduction are highly similar across different control analyses (r = 0.8100 ± 0.1603, mean ± std). The surface maps of different cases are shown in the supplementary Figure S15 and S16. Only the upper triangle of the matrix is shown.

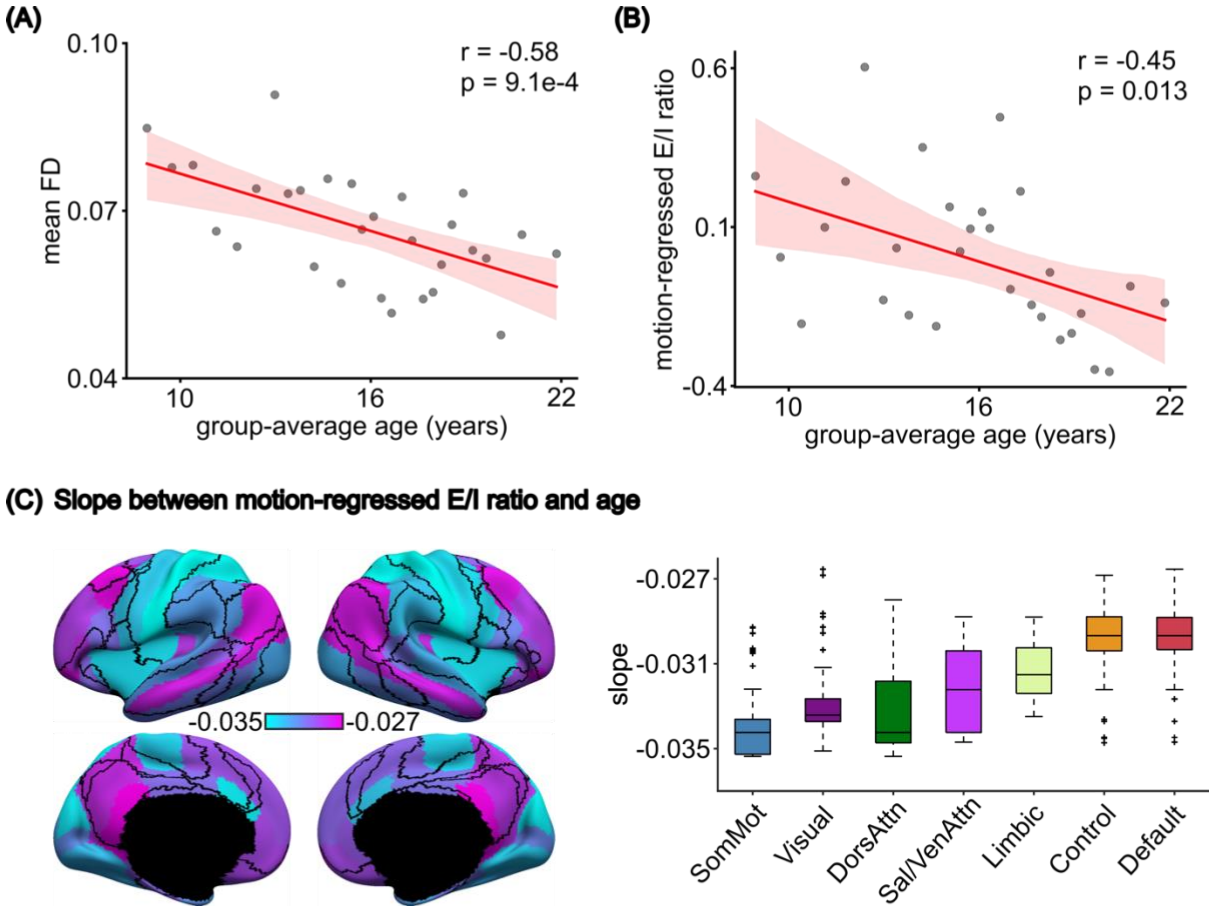

Figure S18. (A) Mean framewise displacement (FD) decreased with age (*r* = -0.58, *p* = 9.1e-4). (B) After regressing out mean FD from the estimated E/I ratio across all age groups, the residuals still significantly decreased with age (*r* = -0.45, *p* = 0.013). (C) Spatial distribution of linear regression slope between FD-regressed E/I ratio and age. All slopes were negative and significant (FDR $q < 0.05$). (D) The slopes exhibited a spatial gradient with sensory-motor networks showing the fastest reduction and association networks showing slower reduction. The boxes show the inter-quartile range (IQR) and the median. Whiskers indicate 1.5 IQR. Black crosses represent outliers.

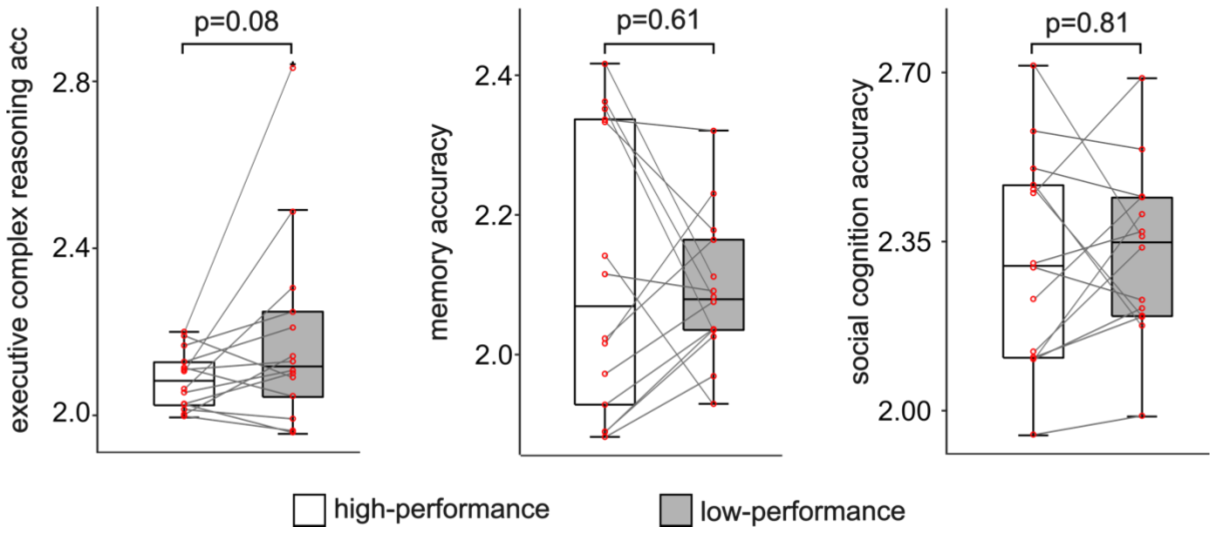

Figure S19. Box plots of E/I ratio estimated from the three domain-specific accuracy scores from Penn Computerized Neurocognitive Battery (CNB). For each 1-year interval, participants whose age are within this interval are extracted to form one age group. For each age group and cognitive score, participants with cognitive scores above the median are assigned to a high-performance group, the rest are assigned to a low-performance group. Both high- and low-performance groups are further divided into subgroups. Participants within each subgroup are randomly assigned to training and validation sets. Each box plot shows the E/I ratio of high- and low-performance group associated with each cognitive score. We observed no significant difference between E/I ratio of high- and low-performance groups for any of the three cognitive scores. The boxes show the inter-quartile range (IQR) and the median. Whiskers indicate 1.5 IQR.

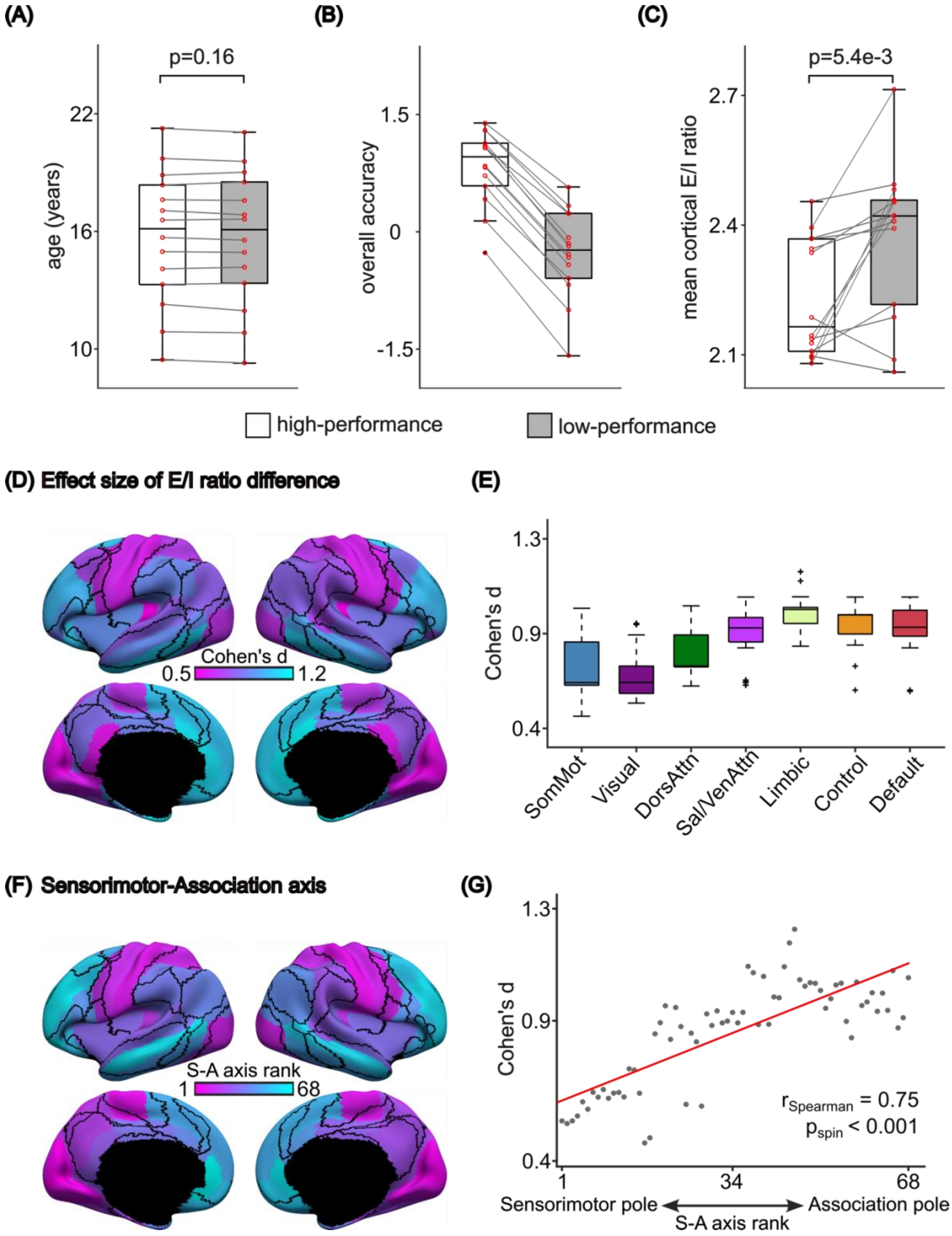

Figure S20. PNC cognition analysis results obtained from 4 additional training-validation participants splits (Figure. S20 to S23). To generate a training-validation split, 885 PNC participants were sorted according to age in an ascending order. For each 1-year interval, participants whose age are within this interval are extracted to form one age group. For each age group, participants with cognitive scores above the median are assigned to a high-performance group, the rest are assigned to a low-performance group. Both high- and low-performance groups are further divided into 14 subgroups. Participants within each subgroup

are randomly assigned to training and validation sets. The random participant splits and analyses were repeated 5 times. (A) Boxplots of age, (B) 'Overall accuracy', and (C) Mean cortical E/I ratio of high- and low-performance groups. (D) Spatial distribution of effect size of regional E/I ratio difference between high-performance and low-performance groups. (E) On a network level, the effect sizes of E/I ratio differences follow a hierarchical structure. We chose the split that had the highest median correlation of regional Cohen's d values with the other 4 splits as the most representative split and showed in the main result section. The results of the 4 splits are consistent with our main results. The boxes show the inter-quartile range (IQR) and the median. Whiskers indicate 1.5 IQR. (F) ROI rankings along the sensorimotor-association (S-A) axis. Lower ranks were assigned to ROIs that were more towards the sensorimotor pole; higher ranks were assigned to ROIs that were more towards the association pole. (G) Agreement between the effect size of E/I ratio difference and S-A axis rank.

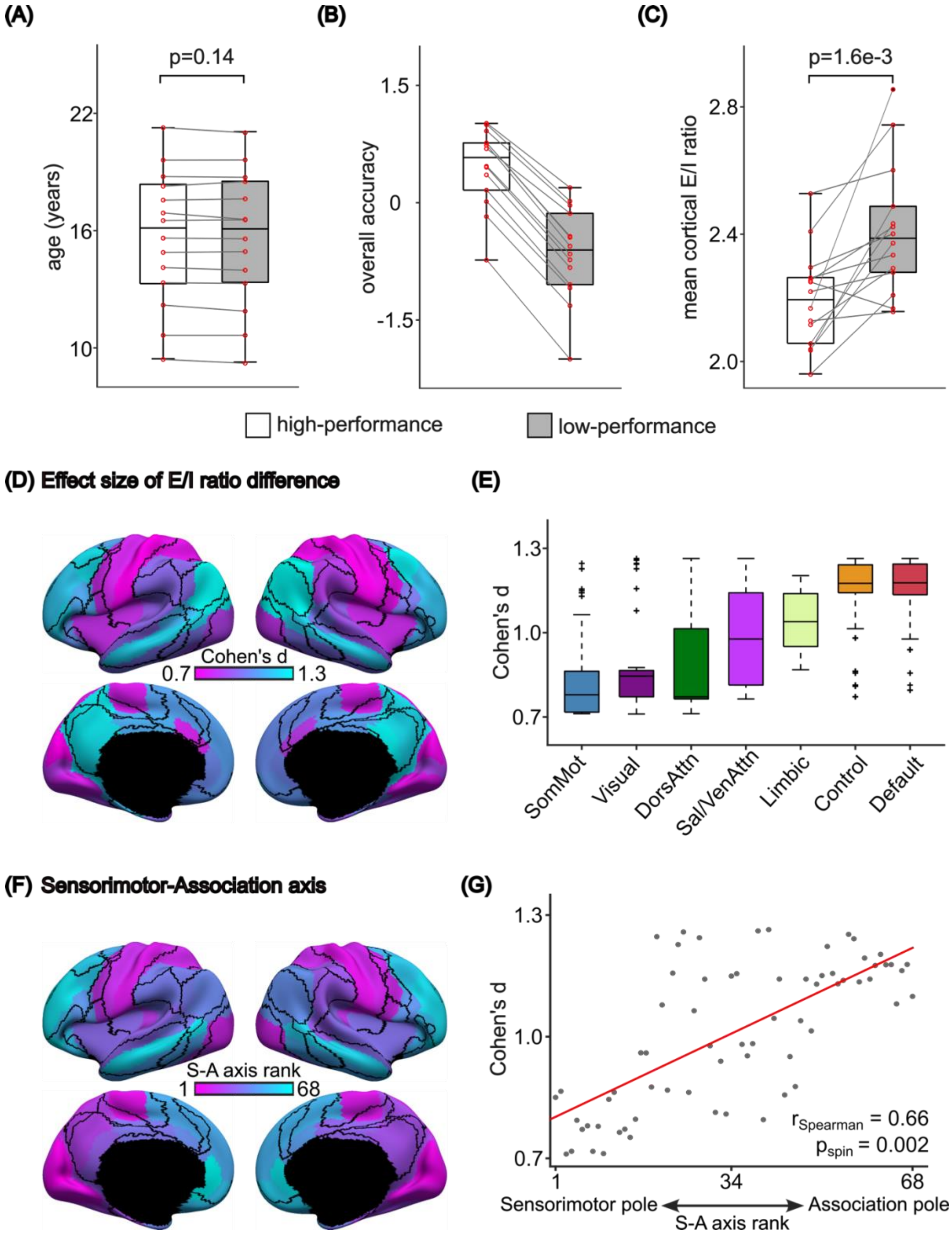

Figure S21. The 3rd training-validation participant split for PNC cognition analysis.

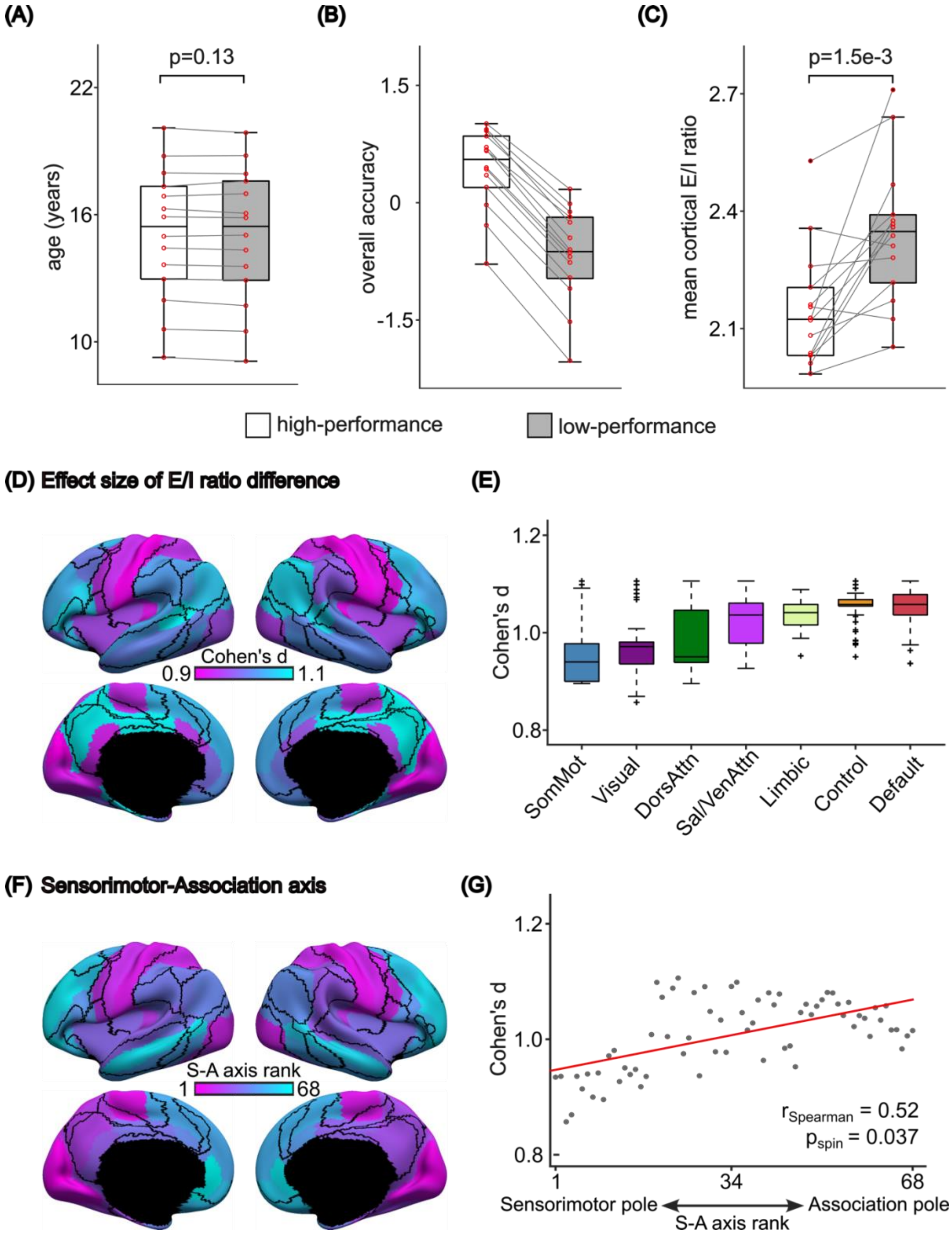

Figure S22. The 4th training-validation participant split for PNC cognition analysis.

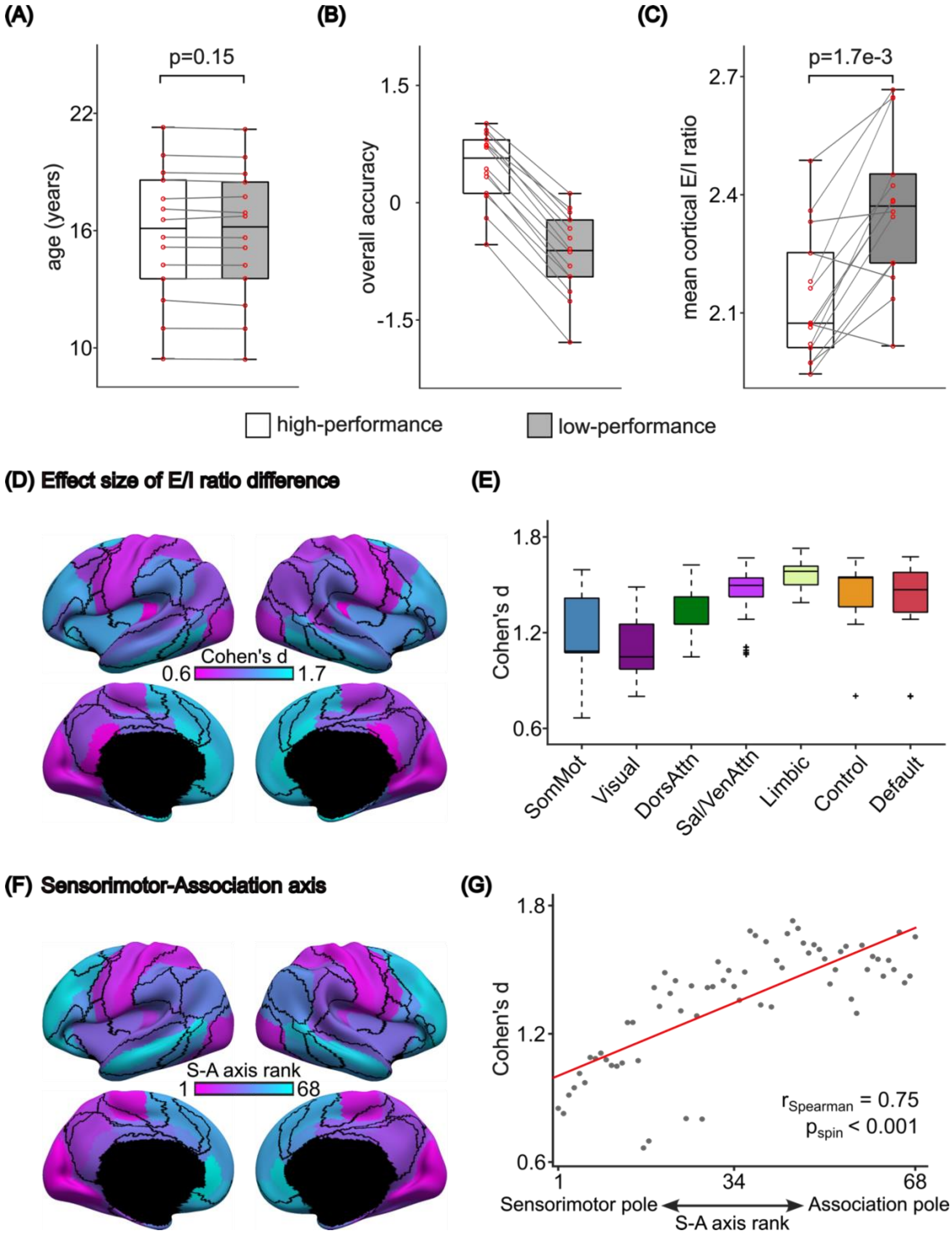

Figure S23. The 5th training-validation participant split for PNC cognition analysis.

	split #1	split #2	split #3	split #4	split #5
split #1	1	0.7760	0.8784	0.8339	0.7057
split #2		1	0.4565	0.6741	0.9754
split #3			1	0.7793	0.3456
split #4				1	0.6247
split #5					1

Figure S24. Pairwise correlation of regional effect sizes (i.e., Cohen's d) between E/I ratios of high- and low-performance groups across 5 participant splits (split #1-5). For each split, high- and low-performance groups were divided into 14 subgroups with training and validation sets. The spatial distributions of the rate of E/I ratio reduction are highly similar across the 5 splits (r = 0.7050 ± 0.1911, mean ± std). Split #1 corresponds to the results shown in Figure 5 of the main text. The surface maps of different splits are shown in the main result (Figure 5D) and supplementary information (Figure. S20 to S23). Only the upper triangle of the matrix is shown.

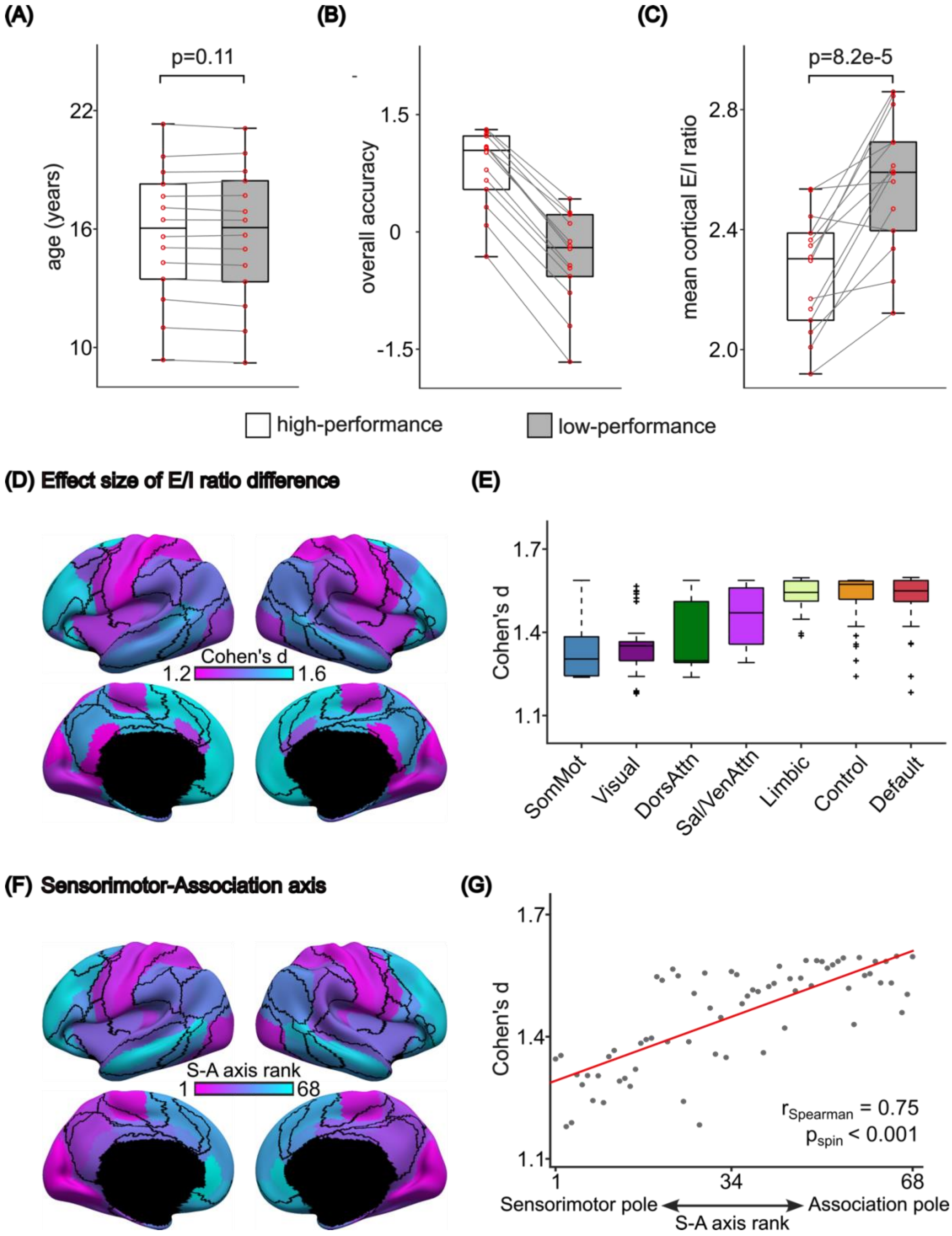

Figure S25. PNC cognition analysis results with relaxed excitatory firing rates thresholds. This figure is similar to Figure 5 but with the acceptable excitatory firing rates range was set to be less strict (2.5Hz to 3.5Hz). (A) Boxplots of age, (B) 'Overall accuracy', and (C) Mean cortical E/I ratio of high- and low-performance groups. (D) Spatial distribution of effect size of regional E/I ratio difference between high-performance and low-performance groups. (E) Box plot of Cohen's d of vertex-level E/I ratio differences grouped by 7 resting-state networks. The boxplots comprised values obtained by "transferring" the parameter estimates

from the 68 Desikan parcels to all vertices (from the underlying cortical meshes) comprising each anatomical parcel. The vertex wise parameter values were then segregated based on the seven resting-state networks. Therefore, there were 3203, 2478, 1523, 1520, 1067, 1438 and 2886 values comprising the boxplots for somatomotor, visual, dorsal attention, ventral attention, limbic, control and default networks respectively. The boxes show the inter-quartile range (IQR) and the median. Whiskers indicate 1.5 IQR. Black crosses represent outliers. The difference of E/I ratio between high- and low-performance groups follows a hierarchical structure. Cohen's d of E/I ratio differences in cognition is larger in association regions compared to sensory regions. (F) ROI rankings along the sensorimotor-association (S-A) axis. Lower ranks were assigned to ROIs that were more towards the sensorimotor pole; higher ranks were assigned to ROIs that were more towards the association pole. (G) Agreement between the effect size of E/I ratio difference and S-A axis rank.

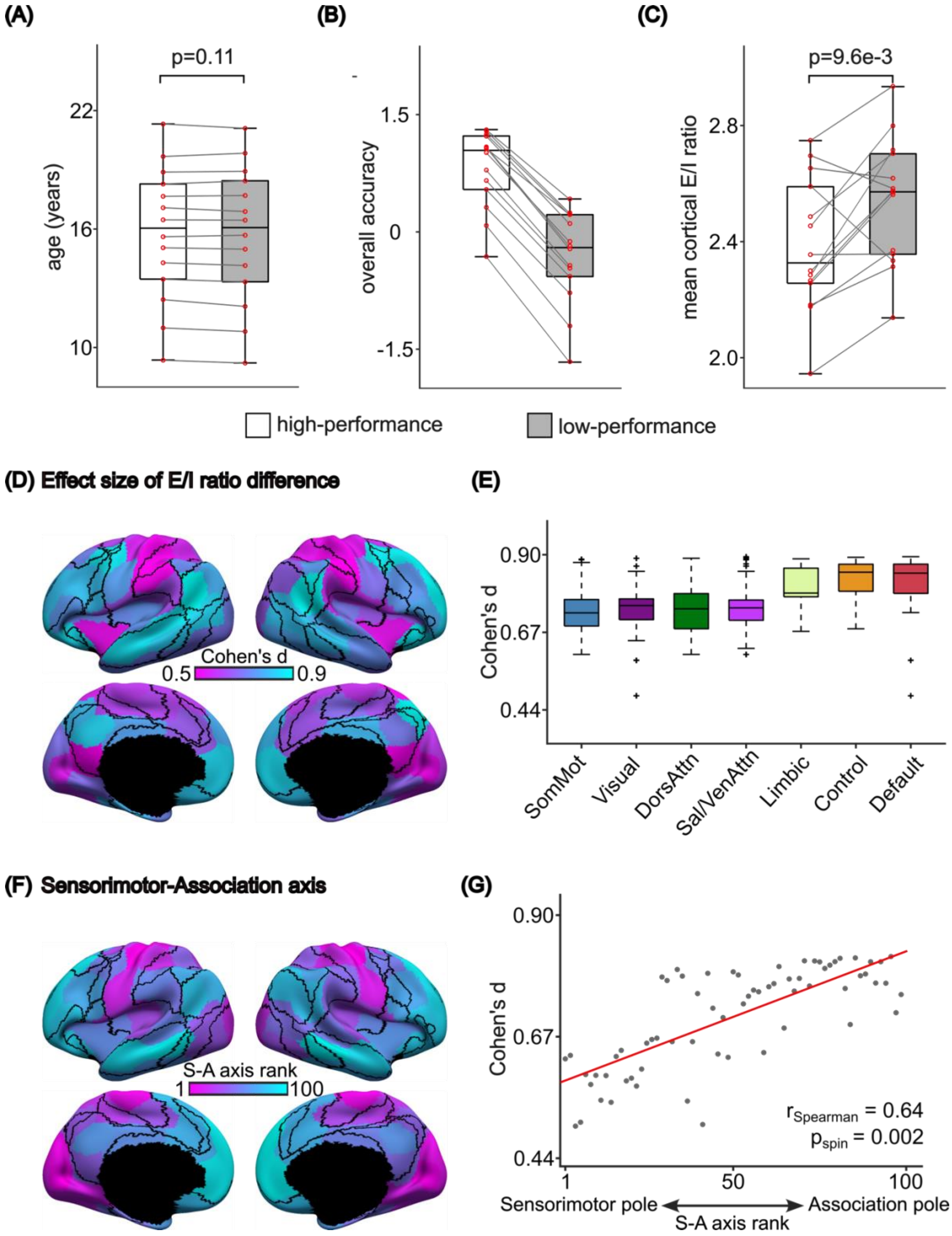

Figure S26. PNC cognition analysis results in Yan 100-ROI parcellation. This figure is similar to Figure 5 but utilizes the Yan 100-ROI parcellation with symmetric left and right hemisphere ROIs. (A) Boxplots of age, (B) ‘Overall accuracy’, and (C) Mean cortical E/I ratio of high- and low-performance groups. (D) Spatial distribution of effect size of regional E/I ratio difference between high-performance and low-performance groups. (E) Box plot of Cohen’s d of vertex-level E/I ratio differences grouped by 7 resting-state networks. The boxplots comprised values obtained by “transferring” the parameter estimates from the 100

Yan parcels to all vertices (from the underlying cortical meshes) comprising each anatomical parcel. The vertex wise parameter values were then segregated based on the seven resting-state networks. Therefore, there were 3203, 2478, 1523, 1520, 1067, 1438 and 2886 values comprising the boxplots for somatomotor, visual, dorsal attention, ventral attention, limbic, control and default networks respectively. The boxes show the inter-quartile range (IQR) and the median. Whiskers indicate 1.5 IQR. Black crosses represent outliers. The difference of E/I ratio between high- and low-performance groups follows the sensory-to-association (SA) axis. Cohen's d of E/I ratio differences in cognition is larger in association regions compared to sensory regions. (F) ROI rankings along the sensorimotor-association (S-A) axis. Lower ranks were assigned to ROIs that were more towards the sensorimotor pole; higher ranks were assigned to ROIs that were more towards the association pole. (G) Agreement between the effect size of E/I ratio difference and S-A axis rank.

	split #1	relaxed r_E range	Yan
split #1	1	0.9258	0.8002
relaxed r_E range		1	0.7725
Yan			1

Figure S27. Pairwise correlation of regional effect sizes (i.e., Cohen's d) between E/I ratios of high- and low-performance groups across different control analyses based on split #1. Split #1 corresponds to the results shown in Figure 5 of the main text. The spatial distributions of the rate of E/I ratio reduction are highly similar across different control analyses (r = 0.8328 ± 0.0817, mean ± std). Only the upper triangle of the matrix is shown.
